# Peroxisome Proliferator-Activated Receptor Activation in Precision-Cut Bovine Liver Slices Reveals Novel Putative PPAR Targets in Periparturient Dairy Cows

**DOI:** 10.3389/fvets.2022.931264

**Published:** 2022-07-12

**Authors:** Sebastiano Busato, Hunter R. Ford, Alzahraa M. Abdelatty, Charles T. Estill, Massimo Bionaz

**Affiliations:** ^1^Department of Animal and Rangeland Sciences, Oregon State University, Corvallis, OR, United States; ^2^Department of Nutrition and Clinical Nutrition, Faculty of Veterinary Medicine, Cairo University, Giza, Egypt; ^3^College of Veterinary Medicine, Oregon State University, Corvallis, OR, United States

**Keywords:** PPAR, nutrigenomics, PCLS, dairy cows, liver, peripartum

## Abstract

Metabolic challenges experienced by dairy cows during the transition between pregnancy and lactation (also known as peripartum), are of considerable interest from a nutrigenomic perspective. The mobilization of large amounts of non-esterified fatty acids (**NEFA**) leads to an increase in NEFA uptake in the liver, the excess of which can cause hepatic accumulation of lipids and ultimately fatty liver. Interestingly, peripartum NEFA activate the Peroxisome Proliferator-activated Receptor (**PPAR**), a transcriptional regulator with known nutrigenomic properties. The study of PPAR activation in the liver of periparturient dairy cows is thus crucial; however, current *in vitro* models of the bovine liver are inadequate, and the isolation of primary hepatocytes is time consuming, resource intensive, and prone to errors, with the resulting cells losing characteristic phenotypical traits within hours. The objective of the current study was to evaluate the use of precision-cut liver slices (**PCLS**) from liver biopsies as a model for PPAR activation in periparturient dairy cows. Three primiparous Jersey cows were enrolled in the experiment, and PCLS from each were prepared prepartum (−8.0 ± 3.6 DIM) and postpartum (+7.7± 1.2 DIM) and treated independently with a variety of PPAR agonists and antagonists: the PPARα agonist WY-14643 and antagonist GW-6471; the PPARδ agonist GW-50156 and antagonist GSK-3787; and the PPARγ agonist rosiglitazone and antagonist GW-9662. Gene expression was assayed through RT-qPCR and RNAseq, and intracellular triacylglycerol (TAG) concentration was measured. PCLS obtained from postpartum cows and treated with a PPARγ agonist displayed upregulation of *ACADVL* and *LIPC* while those treated with PPARδ agonist had increased expression of *LIPC, PPARD*, and *PDK4*. In PCLS from prepartum cows, transcription of *LIPC* was increased by all PPAR agonists and NEFA. TAG concentration tended to be larger in tissue slices treated with PPARδ agonist compared to CTR. Use of PPAR isotype-specific antagonists in PCLS cultivated in autologous blood serum failed to decrease expression of PPAR targets, except for *PDK4*, which was confirmed to be a PPARδ target. Transcriptome sequencing revealed considerable differences in response to PPAR agonists at a false discovery rate-adjusted *p*-value of 0.2, with the most notable effects exerted by the PPARδ and PPARγ agonists. Differentially expressed genes were mainly related to pathways involved with lipid metabolism and the immune response. Among differentially expressed genes, a subset of 91 genes were identified as novel putative PPAR targets in the bovine liver, by cross-referencing our results with a publicly available dataset of predicted PPAR target genes, and supplementing our findings with prior literature. Our results provide important insights on the use of PCLS as a model for assaying PPAR activation in the periparturient dairy cow.

## Introduction

Dairy cows experience drastic metabolic challenges during the peripartum, the period encompassing 3 weeks before to 3 weeks after calving. A decrease in feed intake, combined with a sharp increase in energy demands dictated by the rapidly changing metabolic landscape, result in a state of energetic deficiency. This status is counterbalanced by the mobilization of non-esterified fatty acids (**NEFA**) that are used as energy (1, 2). At the crux of these metabolic changes is the liver, as the central organ for gluconeogenesis and lipid metabolism, contributing to the maintenance of energy homeostasis (3). In particular, during the early postpartum hepatic uptake and metabolism of NEFA sharply increase (4). In the liver, absorbed NEFA are either oxidized or esterified into triacylglycerols (**TAG**) that are stored in the tissue or secreted back into circulation through very low density lipoproteins (**VLDL**) (4). Ruminants, and particularly cattle, are biologically predisposed to secrete TAG in VLDL at a lower rate than other species (5); consequently, increases in circulating NEFA can result in steatosis in the liver, significantly limiting hepatic function at a time when it is the most crucial (6). Further, partial oxidation of NEFA leads to the production of ketones, including β-hydroxybutyrate, supraphysiological levels of which (>3.0 mmol/L) lead to clinical ketosis, with detrimental effects on the animal (7). Thus, it is not surprising that studies on the biology of the periparturient dairy cow focus particularly on liver activity, metabolites, and liver-specific pathways.

Perhaps counterintuitively, evidence shows that energy restriction in prepartum improves the ability of the liver to cope with postpartum stressors, leading to lower postpartum NEFA, total hepatic lipids and TAG, when compared to overfed cows (8), or cows fed *ad libitum* (9). From a molecular standpoint, prepartum feed restriction in dairy cows leads to greater hepatic NEFA uptake and intracellular transport postpartum (10), increases gluconeogenic capacity (11, 12), as well as the expression of genes involved in lipid metabolism (11). Early regulation of pathways related to energy homeostasis and lipid metabolism in the liver may “prime” the organ, and lead to a quicker metabolic response in the early postpartum, contributing to the underrepresentation of pathophysiological conditions; in this context, the role of the Peroxisome Proliferator-activated Receptors (PPAR) could be crucial (13).

PPAR are a group of transcriptional regulators that belong to the nuclear receptor superfamily, of which three isotypes are known and characterized: PPARα, PPARδ, and PPARγ (14). In bovine, expression of PPARα is detected primarily in the liver, while PPARγ is highly abundant in the adipose tissue, and PPARδ is rather ubiquitously expressed (13, 15). PPAR activity, dependent on intracellular concentration of suitable ligands, is known to be modulated by fatty acids and their metabolites (14). Broadly speaking, genes that were identified as PPAR targets code for proteins involved in fatty acid metabolism in the liver, lipid catabolism and insulin sensitivity in the adipose tissue, and in regulation of inflammation and the immune response (13). Crucially, some of the genes upregulated by energy restriction and/or the transition from pregnancy to lactation are targets of PPAR (16), which suggest a strong involvement of PPAR in the hepatic response to metabolic changes in the peripartum. Recently our group showed that, in immortalized mammary, liver, and endothelial bovine cells, PPAR activity is strongly induced by NEFA present in blood serum of early lactation Jersey cows (17). Our results support the hypothesis that a moderate increase in circulating NEFA prepartum, brought forth by energy restriction, improves hepatic fitness postpartum through activation of PPAR.

The study of hepatic metabolism and gene expression *in vitro* can aid in the quantification of parameters of interest with remarkable precision. Currently, the gold standard for cell-based hepatic studies is the isolation and purification of parenchymal cells (hepatocytes) using a two-step perfusion method on either whole liver or the caudate lobe alone (18). While feasible in smaller species, whole-liver or single-lobe isolation of hepatocytes in livestock remains incredibly impractical, with relatively low viability and rapid phenotype loss in culture (19, 20). An alternative can be the use of hepatic cells isolated from newborn calves (21–24); however, extrapolation of results to hepatic metabolism of periparturient dairy cows is problematic, as from a biological standpoint liver of newborn calves is radically different than the liver of an adult cow (25).

A valuable alternative could be the use of precision-cut liver slices (PCLS), obtained through precise dissection of cylindrical liver samples under conditions that facilitate cell survival and allow maintenance of tissue morphology. As demonstrated in other species, PCLS can be a valuable tool to estimate hepatic lipid metabolism (26) and transcriptomic changes (27) *ex vivo*. However, the adoption of PCLS as a research tool for ruminants remains low. To the best of our knowledge, no published manuscript utilizes PCLS culture to study whole-transcriptome changes in the bovine liver.

The objective of the present study was to evaluate the feasibility of using PCLS obtained from periparturient dairy cows to assess activation of PPAR *via* transcriptomics-based approaches and identify PPAR target genes in bovine. We hypothesize that culture and treatment of PCLS with known PPAR agonists and antagonists would result in measurable changes in gene expression, the identification of which could shed light on the consequences of greater PPAR activation in the peripartum.

## Materials and Methods

### Animals and Collection of Blood and Liver Tissue

Experimental procedures used in this study were approved by the Institutional Animal Care and Use Committee (IACUC) of Oregon State University (protocol# 4894). Liver biopsies were performed on four primiparous Jersey cows; however, one cow had to be removed from the study (see RT-qPCR). The biopsy was performed both during prepartum (−8.0 ± 3.6 DIM, henceforth referred to as “−10 DIM”) and postpartum (+7.7 ± 1.2 DIM, henceforth referred to as “+10 DIM”). The area selected for puncture was clipped, and decontaminated using povidone iodine medical scrub (055478, Covetrus, OH, USA) followed by a solution of 75% ethanol, applied with a surgical gauze (100-1444, Henry Schein, NY, USA). A small incision was made using a #10 surgical blade (327-1504, Integra Miltex, PA, USA), and a 6 mm i.d. trocar was used to collect liver tissue up to 3 times until sufficient tissue was obtained (~500–800 mg). The tissue was immediately transferred to a sterile tissue culture dish (351029, Corning Falcon, NY, USA), rinsed immediately in sterile phosphate buffered saline (25-508P, Genclone, CA, USA), and transferred to a 50 mL conical tube containing ice-cold Krebs-Henseleit buffer [KHB, prepared as previously described (28)] until further processing. Liver samples were transported from the collection site to the laboratory within 1 h. Pre-prandial blood samples were collected from each animal immediately before the liver biopsy, using Vacutainer blood collection tubes without anti-coagulants (366430, Becton, Dickinson and Company, NJ, USA). Samples were allowed to coagulate at room temperature for no <30 min. The serum was separated by centrifugation (15 min, 1,500 × g, 25°C), and kept at room temperature until all the liver slices were prepared (<1 h).

### PCLS Preparation and Culture

PCLS were prepared following the protocol developed by De Graaf and collaborators (28), with few modifications. Briefly, liver tissue samples were transported to the laboratory in ice-cold KHB; upon arrival, they were immediately embedded in low temperature gelling ultrapure agarose (16500-100, Invitrogen, CA, USA) inside an 8 mm mold-plunger assembly (MD2200, tissue embedding unit, Alabama Research & Development, AL, USA). The plunger with the embedded tissue was then transferred to a Krumdieck Tissue Slicer (MD1000-A1, Alabama Research & Development, AL, USA), pre-filled with ice-cold KHB; slice thickness was set at 280–300 μm, with a cycle speed of ~35 and using the “intermittent blade mode”. Slices were then collected and placed on a new tissue culture dish, prefilled with a minimum amount of cold KHB to prevent dehydration. A total of 36 PCLS were selected for each animal at each timepoint, evaluating visually to identify those with the a clear circular shape, and without holes or patent morphological irregularities. The 36 PCLS were thus transferred to three 12-well culture plates (665180, Greiner Bio One, NC, USA) and the treatments were applied in duplicates. In Plates 1 and 2, PCLS were cultured in William's Medium E (WME, A1217601, Gibco, Thermo Fisher Scientific, MA, USA), supplemented with GlutaMAX (35050-061, Gibco, NY, USA), 14 mM of D-glucose monohydrate (0643-1KG, VWR, OH, USA), and 50 μg/mL of gentamycin (15750060, Life Technologies, OR, USA). Within the plate, PCLS were treated with 100 μM of the PPARα agonist WY-14643 (70730, Cayman Chemicals, MI, USA), 50 μM of the PPARδ agonist GW-501516 (ALX-420-032-M005, Enzo, NY, USA), or 100 μM of the PPARγ agonist rosiglitazone (R0106, TCI, OR, USA); 200 μM of palmitic acid (100905, MP Biomedicals, CA, USA) or 100 μM of serum NEFA, isolated *via* solid phase extraction as previously described (17). Palmitate was supplied unbound from albumin to mimic a local concentrated release (17). WY-14643 concentration was selected based on prior reports (29); additionally, according to our findings in an immortalized model of bovine liver (17), the dose-dependent response to PPARα and PPARγ was similar (hence the 100 μM concentration of both WY and rosiglitazone), while PPARδ modulation was about twice as sensitive (hence the 50 μM dose for GW-501516). Additionally, peak PPAR activation for palmitic acid was achieved at 200 μM. The concentration of NEFA was chosen to mimic the physiological concentration of NEFA in the lactating dairy cow. In Plate 3, PCLS from each cow were cultured in blood serum isolated from the same cow on the day of the liver biopsy; PPAR antagonists were added at 50 μM in duplicates to the wells: for PPARα, GW-6471 (9453, CAS# 880635-03-0, BioVision incorporated, CA, USA); for PPARδ, GSK-3787 (3961/10, CAS# 188591-46-0, Tocris, Bio-Techne Corporation, MN, USA); for PPARγ, GW-9662 (70785, CAS# 22978-25-2, Cayman Chemicals, MI, USA). Additionally, palmitic acid and serum NEFA were also supplemented with doses as in Plates 1 and 2. All treatments were adjusted for the vehicle (DMSO, D2438, Millipore Sigma, MO, USA) at a volume of 0.8%. Plates were placed in a modular incubator chamber (MIC-101, Billups-Rothenberg, CA, USA), clamped shut, and flushed with carbogen (95% O2, 5% CO2) for 10 min. The modular chamber was placed inside a cell culture incubator, and atop a benchtop orbital shaker located inside of the incubator. The slices were incubated for 18 h at 37°C, ~100 rpm.

### TAG Quantification

PCLS from Plate 1 were collected, and the two replicates for each treatment were pooled. Slices were homogenized using a handheld tissue homogenizer (850101019999, Scilogex, CT, USA), and whole-homogenate TAG were measured according to the manufacturer's instructions (10010303, Cayman Chemicals, MI, USA). A small amount of tissue homogenate from the first dilution was retained separately, and protein concentration was assessed using a Pierce BCA Protein Assay Kit (23225, Thermo Scientific, MA, USA), following the manufacturer's instructions. Assayed TAG concentration was normalized to the protein concentration.

### RNA Sequencing and RT-qPCR

#### RNA Isolation

PCLS from Plates 2 and 3 were collected after incubation, and transferred to separate screw-cap vials (490003-520, VWR, PA, USA), pre-filled with 600 μL of ice-cold TRIzol reagent (15596026, Thermo Scientific, MA, USA) and two 3.2 mm beads, homogenized using a Geno/Grinder Automated Tissue Homogenizer (2010-115, SPEX SamplePrep, NJ, USA; courtesy of the Department of Crop and Soil Sciences, Oregon State University, OR, USA). The tissue was disrupted in short burst (45 s) at 1,500 rpm, followed by incubation on ice for 3 min; the disruption-incubation cycle was repeated up to three times, or until no tissue pieces were visible within the tubes. Immediately after disruption, 120 μL of pre-chilled chloroform was added to the tube, and the samples were mixed by inverting the tube, and incubated on ice for 5 min. After incubation, the samples were transferred to a new 1.7 mL microcentrifuge tube, and centrifuged at 4°C for 15 min, 15,000×g. The upper-phase supernatant (~200 μL) was collected and RNA was purified using a Mag-MAX-96 Total RNA Isolation Kit (AM1830, Invitrogen, MA, USA) following the manufacturer's instructions, with minor modifications: briefly, 100 μL of each sample was transferred to the first row (A) of a 96-Well DeepWell Storage Plate (260251, Thermo Scientific, MA, USA). Rows B-F contained reagents supplied with the kit: 20 μL of the magnetic beads mix (row B), 150 μL of wash solution 1 (row C), 150 μL of wash solution 2 (row D), and 50 μL of DNAse-RNAse free water (VWRL0201-0500, VWR, PA, USA) in rows E and F. A suitable protocol was then generated to mimic the manufacturer's protocol with a KingFisher Duo Purification System (5400110, Thermo Scientific, MA, USA). Eluted RNA was measured using a SpectraDrop Micro-Volume Microplate in a SpectraMax plus 384 spectrophotometer (89212-396, Molecular Devices, CA, USA). Average 260/230 and 260/280 ratios were 2.06 ± 0.24 and 1.69 ± 0.27, respectively. RNA integrity was assessed by the Center for Genome Research and Bioinformatics at Oregon State University using an Agilent Bioanalyzer 2100 (G2939BA, Agilent, CA, USA). For one animal, the RIN for the +10 DIM PCLS was below 3. Further, principal component analysis of RNAseq data revealed that animal to be a clear outlier and was removed from the study. Upon removal of that animal, resulting RNA integrity numbers were 7.26 ± 0.64 (mean ± SD).

#### RT-qPCR

Complementary DNA (cDNA) synthesis, PCR, and data analysis using LinRegPCR were performed as previously described (25). Primers used in this study are listed in Supplementary Table S1; all primers not sourced from a prior study were assessed by amplifying a mixture of bovine cDNA, and the resulting amplicon was sequenced by the Center for Genome Research and Bioinformatics (currently Center for Quantitative Life Sciences) at Oregon State University using an ABI 3730 capillary sequencer machine. Amplicons were aligned against the bovine genome using NCBI Basic Local Alignment Search Tool (BLAST) (30) to ensure specificity. Selection of internal control genes was accomplished using GeNorm (31). Potential reference genes *GAPDH, MRPL39* and *UXT* were tested *via* GeNorm. Results from GeNorm indicated that the geometrical mean of those 3 reference genes provided a robust normalization (V2/3 < 0.18).

#### Library Preparation and Sequencing

RNA isolated from PCLS from postpartum animals cultivated in William's E Medium and treated with the three isotype-specific PPAR agonists, as well as the control group, were sent to the Center for Genome Research and Bioinformatics at Oregon State University for high-throughput sequencing. Library construction was obtained using a QuantSeq 3' mRNA-Seq Library Prep Kit FWD for Illumina (015.96, Lexogen, NH, USA). Sequencing was performed on an Illumina HiSeq3000 platform, at 60 samples/lane. The raw reads have been deposited (GEO accession number GSE183063).

#### Quality Control and Differential Gene Expression Analysis

Quality control was assayed using MultiQC v1.8 (32) (https://multiqc.info/). Reads were then trimmed based on PHRED score and adapter presence using TRIMMOMATIC v0.39 (33) (https://github.com/usadellab/Trimmomatic) with arguments LEADING:5 TRAILING:5 SLIDINGWINDOW:4:5 MINLEN:3. A genome index was generated using the ARS-UCD1.2 *Bos Taurus* genome (http://ftp.ensembl.org/pub/release-104/fasta/bos_taurus/dna/) and the ARS-UCD1.2.104 annotation (http://ftp.ensembl.org/pub/release-104/gtf/bos_taurus/), using the genomeGenerate function of STAR v2.7.1 (34) (https://github.com/alexdobin/STAR). Trimmed reads were aligned against the reference genomic index using STAR, and the average overall alignment rate was 85.46%. Aligned.sam files were sorted and converted to.bam using samtools v1.0 (35) (https://github.com/samtools/samtools), and gene count matrices were generated using stringtie v 2.0 (https://ccb.jhu.edu/software/stringtie/).

Differential expression was determined using the DESeq2 package, v1.30.1 (36) (https://bioconductor.org/packages/release/bioc/html/DESeq2.html) in R v3.9, after filtering for low counts (any transcript with raw count ≤ 4). Three contrasts were generated (PPARA agonist vs. CTR, PPARD agonist vs. CTR, and PPARG agonist vs. CTR) and DEG were considered significant with an FDR-adjusted *p*-values below 0.2.

#### Bioinformatics Analyses: Ontology and Function

Functional analysis was performed using the Dynamic Impact Approach (37), as well as DAVID (38) (https://david.ncifcrf.gov/). Figures regarding functional analysis results were generated using the ggplot2 R package v3.3.3 (https://ggplot2.tidyverse.org/reference/ggplot.html), and treemaps using REVIGO (39) (http://revigo.irb.hr/).

To discriminate between actual gene targets of PPAR and genes that are differentially expressed in response to the treatment but not regulated by PPAR, we cross-referenced our results with the publicly available PPARgene database (http://www.ppargene.org/). This resource provides a comprehensive list of 2,683 predicted targets, based on a logistic regression model that utilizes both experimental high-throughput sequencing data and the degree of conservation of the PPAR binding site within the human and mouse genome (40). A prediction score from 0 to 1 is assigned to each gene, with the authors defining a value below 0.6 as “low confidence”, between 0.6 and 0.8 as “medium confidence”, and between 0.8 and 1 as “high confidence”. We extracted only medium and high confidence genes from the dataset to reduce potential confounding factors.

### Statistical Analysis

Normalized RT-qPCR data were log_2_ transformed prior statistical analysis. Data were checked for outliers using PROC REG of SAS and datapoints with a studentized-t >2.8 were removed. Statistical analysis was performed as four separated datasets: PCLS from prepartum cows cultivated in artificial media; PCLS from prepartum cows cultivated in blood serum; PCLS from postpartum cows cultivated in artificial media; and PCLS from postpartum cows cultivated in blood serum. Final datasets were analyzed using PROC GLIMMIX of SAS (v9.4, SAS, NC, USA) using treatment as explanatory variable and cow as random variable using the default covariate model. Postpartum TAG data were analyzed through PROC GLM of SAS (v9.4, SAS, NC, USA), using treatment as the only explanatory variable. In all cases, a *p*-value of 0.1 was considered as the threshold for tendencies and a *p*-value of 0.05 was set as the threshold for significance between the pairwise comparisons.

## Results

### RT-qPCR and TAG Quantification

**Figure 1:**
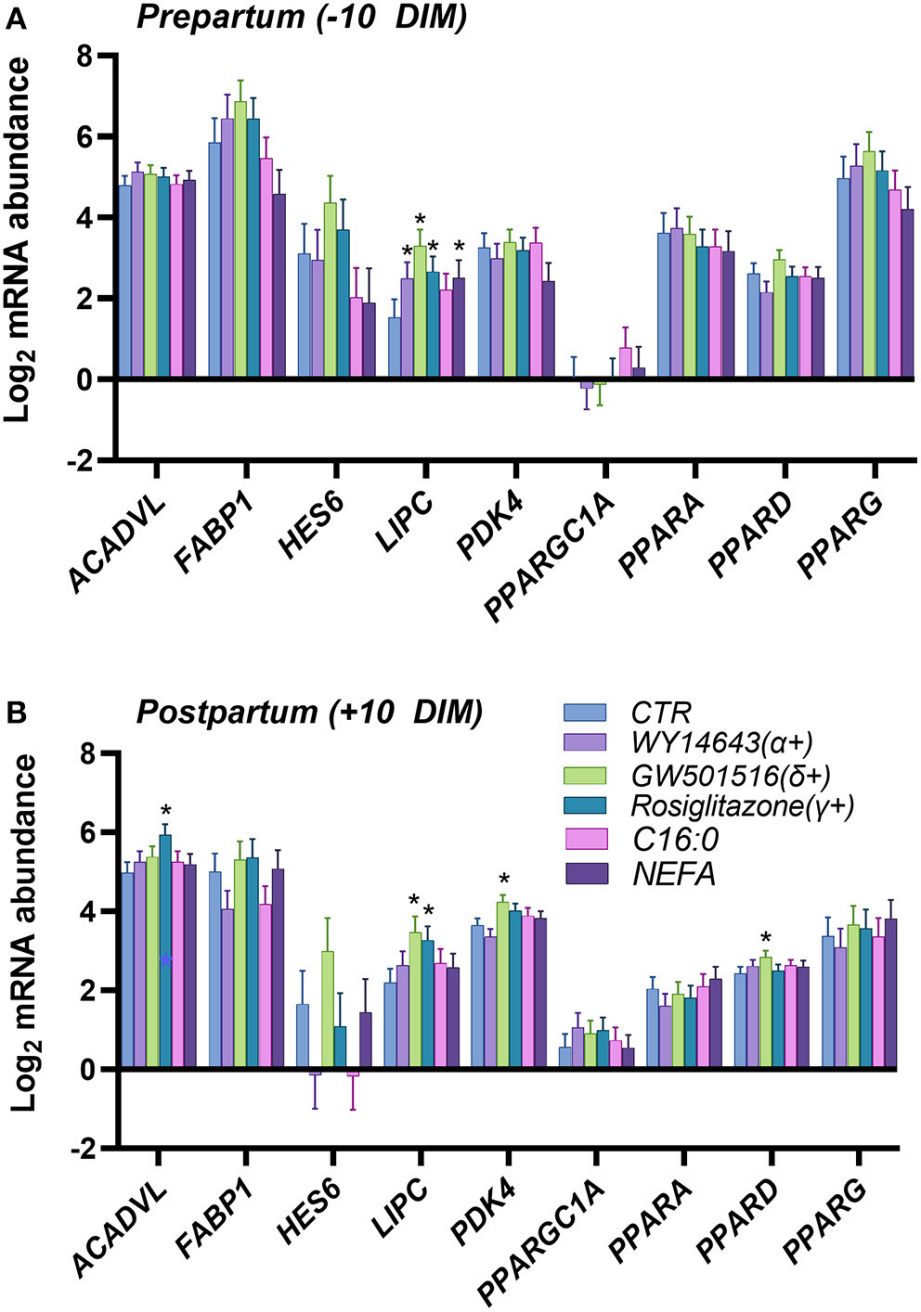
Relative normalized expression of *ACADVL, FABP1, HES6, LIPC, PDK4, PGC1A, PPARA, PPARD* and *PPARG* in response to synthetic PPAR agonists (100 μM WY-14643, a PPARα agonist; 50 μM GW-501516, a PPARδ agonist; and 100 μM Rosiglitazone, a PPARγ agonist), 200 μM palmitic acid, or 100 μM NEFA isolated from each respective animal, in PCLS obtained from prepartum [**(A)**, −10 DIM] and postpartum [**(B)**, +10 DIM] cows, and cultured for 18 h in William's Medium E. *indicates significant differences (*P* < 0.05) when compared to WME control.

**Figure 2:**
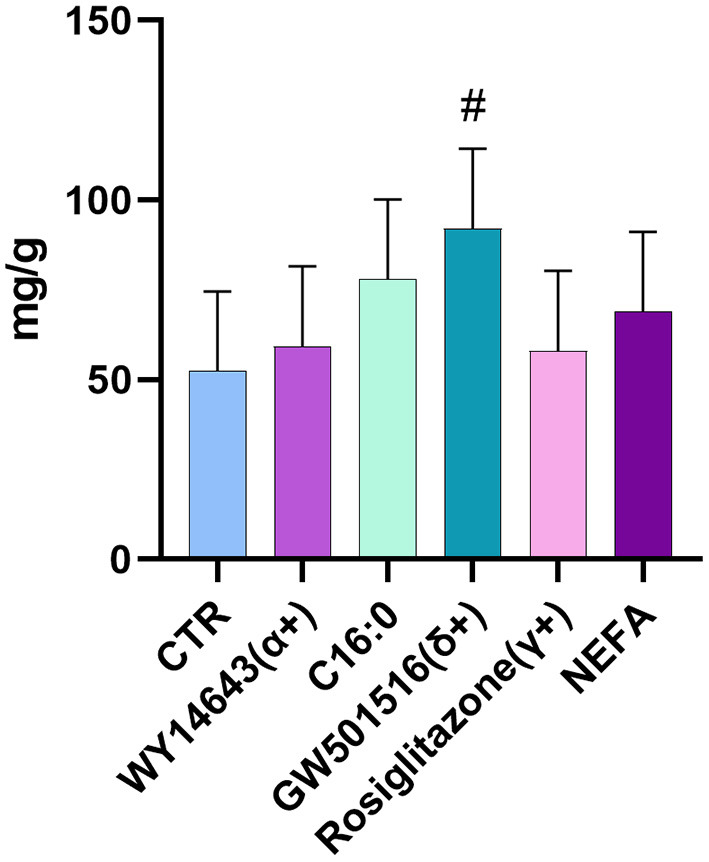
Amount of triacylglycerol in PCLS from postpartum cows (+10 DIM) treated with PPAR isotype-specific agonists (100 μM WY-14643, a PPARα agonist; 50 μM GW-501516, a PPARδ agonist; and 100 μM Rosiglitazone, a PPARγ agonist), 200 μM palmitic acid, or 100 μM NEFA isolated from each respective animal. Results are presented as fold change vs. each animal's control group (PCLS cultured in William's Medium E only). ^#^indicates tendencies (*P* < 0.1) when compared to control. Incubation time was 18 h.

In the first experiment we assessed the transcription of PPAR target genes upon treatment with various PPAR isotypes synthetic agonists in PCLS obtained from pre- and post-partum cows cultivated in synthetic media. In the same experiment we also treated PCLS with C16:0 and NEFA, both previously observed to be agonist of PPAR in bovine cells (17). The PCLS from each cow was treated with NEFA isolated from the same cow. Analysis of relative gene expression through RT-qPCR revealed minimal differences across treatments in the late prepartum (−10 DIM, Figure 1A), as well as the early postpartum (+10 DIM, Figure 1B). In the prepartum, transcription of *LIPC* was increased by all treatments except C16:0, with the highest effect observed for slices treated with a PPARδ agonist (GW-501516; 3.4-fold increase vs. untreated control). In the postpartum, treating liver slices with a PPARγ agonist (rosiglitazone) resulted in 2-fold increased transcription of *ACADVL* vs. untreated control. Treatment with GW-501516 increased expression of *PDK4, LIPC*, and *PPARD*. Only a tendency for higher TAG content in cells was observed in response to PPARδ agonist (Figure 2).

**Figure 3:**
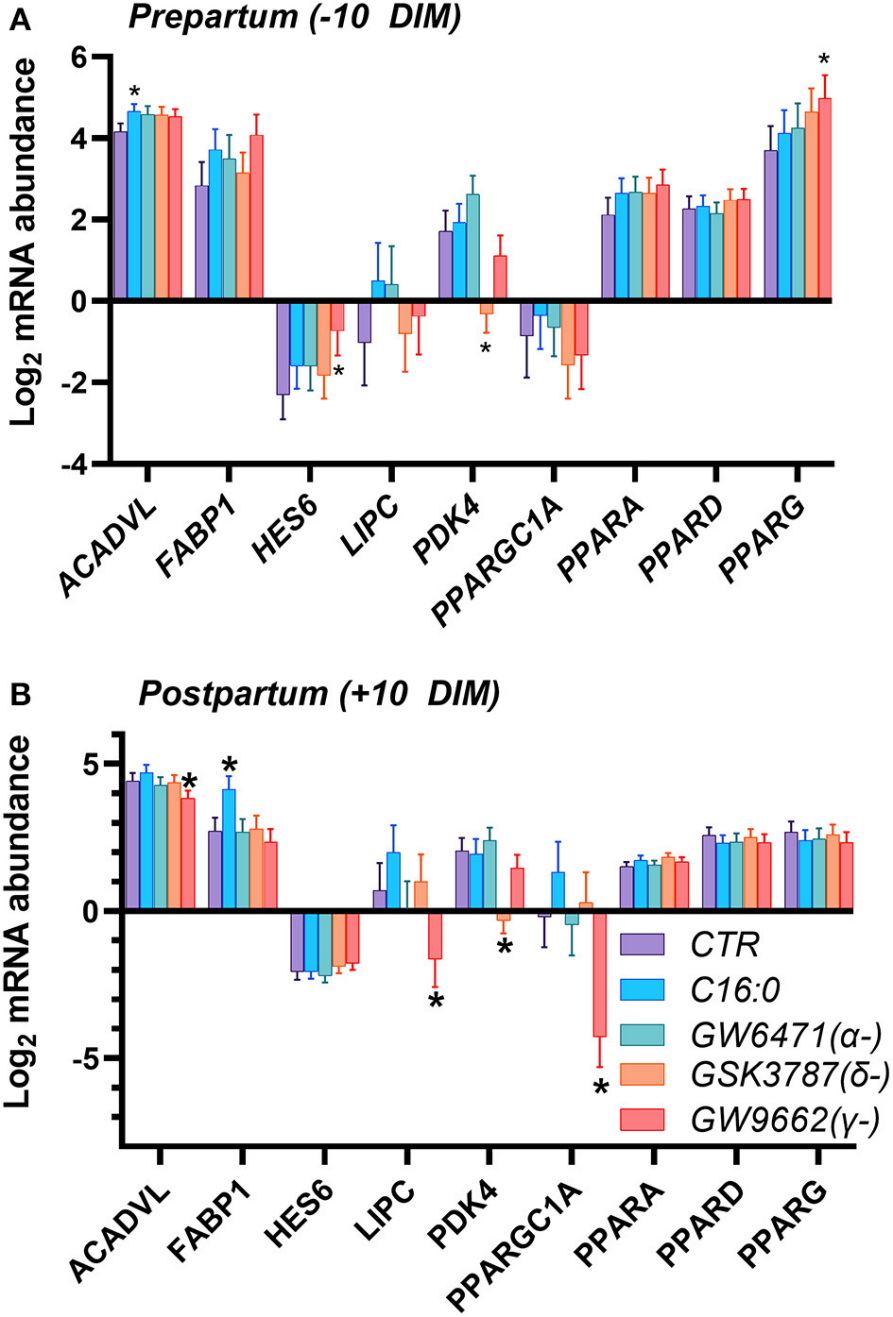
Relative normalized expression of *ACADVL, FABP1, HES6, LIPC, PDK4, PGC1A, PPARA, PPARD* and *PPARG* in response to synthetic PPAR antagonists (50 μM GW-6471, a PPARα antagonist; 50 μM GSK-3787, a PPARδ antagonist; and 50 μM GW-9662, a PPARγ antagonist) or 200 μM palmitic acid, in PCLS obtained from prepartum (**A**, −10 DIM) and postpartum (**B**, +10 DIM) cows, and cultured in blood serum. *Indicates significant differences (*P* < 0.05) when compared to serum control; Incubation time was 18 h.

In a second experiment we assessed which PPAR isotype is activated by blood serum by using various PPAR isotype-specific antagonists with PCLS. Our assumption was that blood serum containing NEFA would activate PPAR. The dose of the antagonists was based on our prior work in immortalized bovine liver cells (17). We also treated cells with C16:0, to mimic supplementation of this fatty acid in live animals. As for the first experiment, we detected a minimal effect on transcription of genes, especially in the PCLS obtained from pre-partum cows. We did not observe any overlap between the two experiments. In the prepartum (Figure 3A), transcription of *ACADVL* was increased in response to C16:0. The addition of the PPARγ antagonist increased the expression of *HES6* and *PPARG*, while the use of the PPARδ antagonist reduced the transcription of *PDK4*. In the postpartum (Figure 3B), treating liver slices with palmitic acid increased expression of *FABP1*, while the PPARδ antagonist significantly downregulated *PDK4* and the PPARγ antagonist decreased transcription of *LIPC* and *PPARGC1A*.

### RNA Sequencing and Functional Analysis

#### Differentially Expressed Genes

Gene expression profiling was performed through RNA sequencing for PCLS from postpartum animals (+10 DIM), which were treated with isotype-specific agonists for PPARα (WY-14643), PPARδ (GW-501516) and PPARγ (rosiglitazone). Complete dataset is available in Supplementary File 1.

Principal component analysis revealed considerable separation with minimal overlap between the treatment groups, suggesting moderate differences in the gene expression landscape (Supplementary Figure S1). Analysis through DESeq2 (Table 1) revealed a total of 140, 173, and 222 DEG with a cut-off of FDR-adjusted *p*-values = 0.1 by the PPARα, PPARδ, and PPARγ agonist, respectively. A more liberal cutoff of FDR-adjusted *p*-values = 0.2 indicated 308, 501, and 379 DEG by the PPARα, PPARδ, and PPARγ agonist, respectively. The latter statistical results were used for downstream analyses.

**Table 1:** List of differentially expressed genes in PCLS for the three comparisons of interest.

**vs. CTR**	**FDR-Adj**	**DEG up**	**DEG down**	**DEG total**
PPARα agonist	0.1	59	81	140
PPARδ agonist	0.1	47	126	173
PPARγ agonist	0.1	127	95	222
PPARα agonist	0.2	191	117	308
PPARδ agonist	0.2	334	167	501
PPARγ agonist	0.2	174	205	379

#### Dynamic Impact Approach

**Figure 4:**
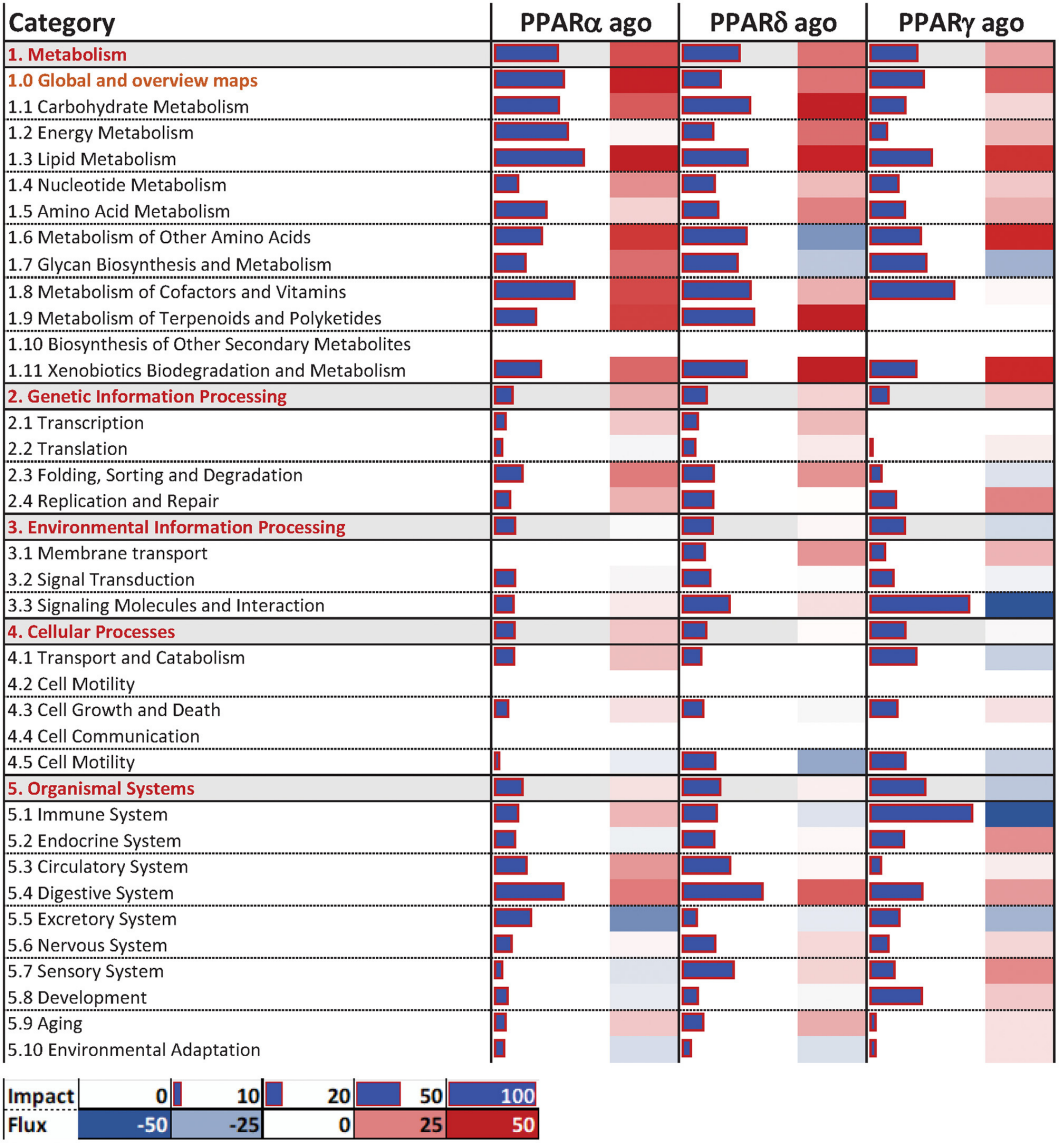
Main categories and subcategories of KEGG pathways induced *via* the use of isotype-specific PPAR agonists in PCLS, as summarized by the Dynamic Impact Approach. Blue bars refer to the impact in terms of overrepresented genes in the pathways, while the shaded cell denotes the flux, i.e., the overall effect on the pathway, with red denoting activation and green denoting inhibition.

The Dynamic Impact Approach (**DIA**) analysis (Figure 4) revealed a large impact of both the PPARα and PPARδ agonists on KEGG categories related to metabolism, chiefly carbohydrate and lipid metabolism, all of which had a pattern toward activation of the pathways. Of note, though the impact of the PPARγ agonist on metabolism was lower than the other two PPAR agonists, a strong effect on lipid metabolism was maintained, also with a positive trend. Additionally, samples treated with the PPARγ agonist displayed a strong inhibitory effect on KEGG subcategories related to “Signaling Molecules and Interaction” and under the “Immune System” subcategory of pathways, while the other two PPAR agonists did not.

**Figure 5:**
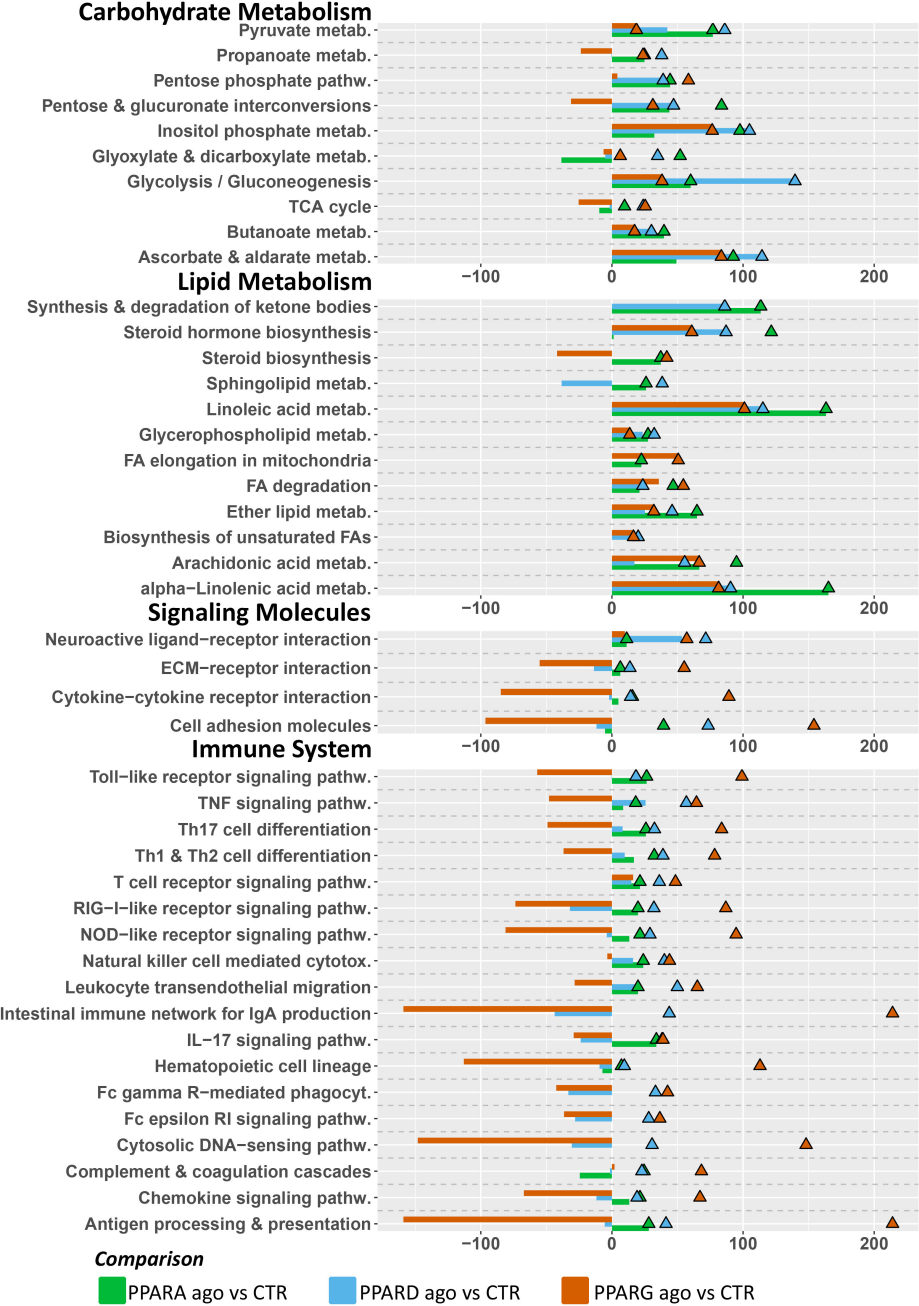
Main KEGG pathways highlighted by DIA, divided by subcategory. Triangles (color-coded) represent the impact in terms of overrepresented genes in the pathways, whereas bars (also color-coded) refer to the overall flux of the pathway, with negative numbers indicating inhibition of the pathway, and positive numbers indicating induction of the pathway.

In terms of individual pathways within the KEGG subcategories (Figure 5), for the “Carbohydrate Metabolism” subcategory, all three groups had a modest positive impact on “Pentose and glucuronate interconversions”, as well as “Glyoxylate and dicarboxylate metabolism”, and “Ascorbate and aldarate metabolism”. In the “Lipid Metabolism” subcategory, PPAR agonists had comparable positive impacts on “Metabolism of linoleic acid”, as well as “Fatty acid degradation” and the “Metabolism of ether lipids” (although to a lesser degree). Additionally, only the PPARα and PPARδ agonists induced the pathway “Synthesis and degradation of ketone bodies”. In the “Signaling Molecules and Interaction” and “Immune System” subcategories of KEGG pathways, the most consistent and noticeable effect was brought forth by the PPARγ agonist, moderately or strongly inhibiting signaling-related pathways such as “Cytokine-cytokine receptor interaction” and “Cell adhesion molecules”. Additionally, signaling of several immune-related receptors like the toll-like receptor (TLR), tumor necrosis factor (TNF), and RIG-I-like receptor and NOD-like receptor was inhibited. Further, the PPARγ agonist inhibited the “Antigen processing and presentation” and “Chemokine signaling” pathways.

#### DAVID

**Figure 6:**
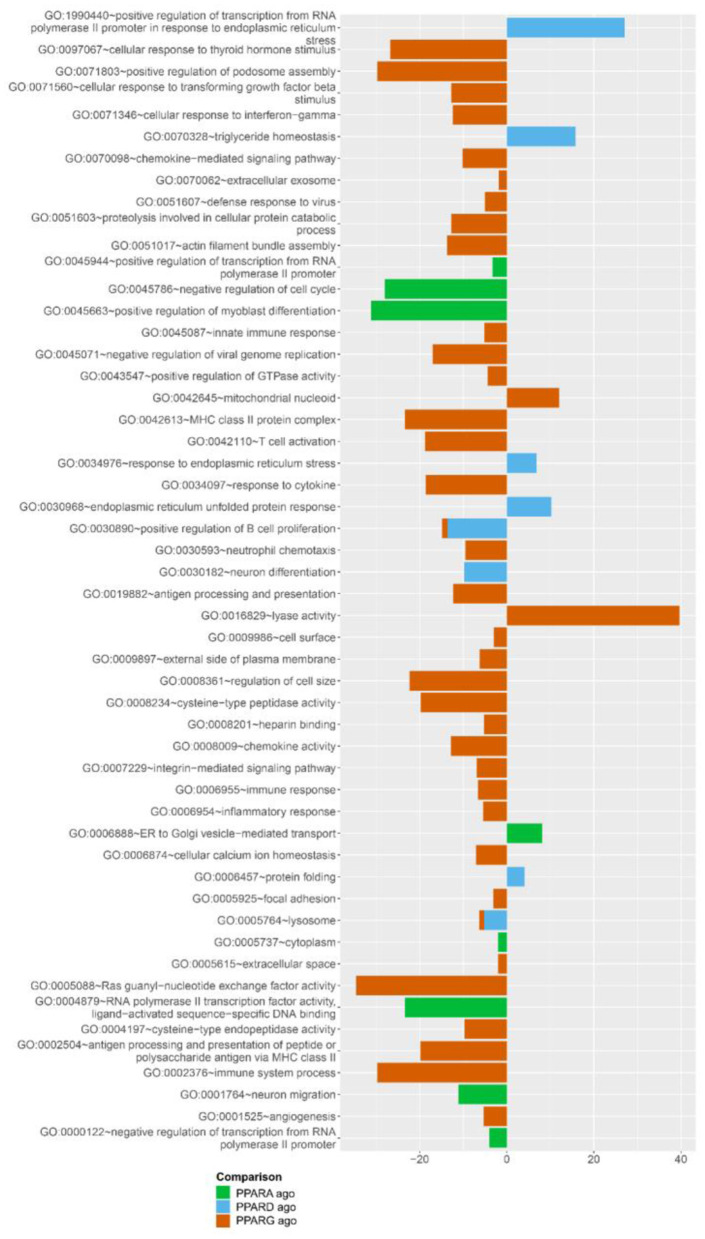
Overrepresented gene ontology (GO) terms with a *p*-value <0.01, according to DAVID, by the three PPAR agonists. Pathways associated with positive numbers are overrepresented in the subset of genes significantly upregulated by the treatment, whereas pathways represented by a negative number are overrepresented in the genes significantly downregulated by the treatment. Absolute numbers correspond to fold enrichment of the pathway.

The analysis of enriched Gene Ontology (GO) terms by DAVID confirmed results from the DIA (Figure 6; Supplementary Figures S2–S7), where samples treated with a PPARγ agonist present an enrichment of the terms “T cell activation,” “response to cytokine,” “chemokine activity,” “immune response”, “inflammatory response,” and “immune system process” within the cohort of downregulated genes. Further, upregulated genes in response to the PPARδ agonist were overrepresented in the “TAG homeostasis” GO term.

### Predicted PPAR Targets

**Figure 7:**
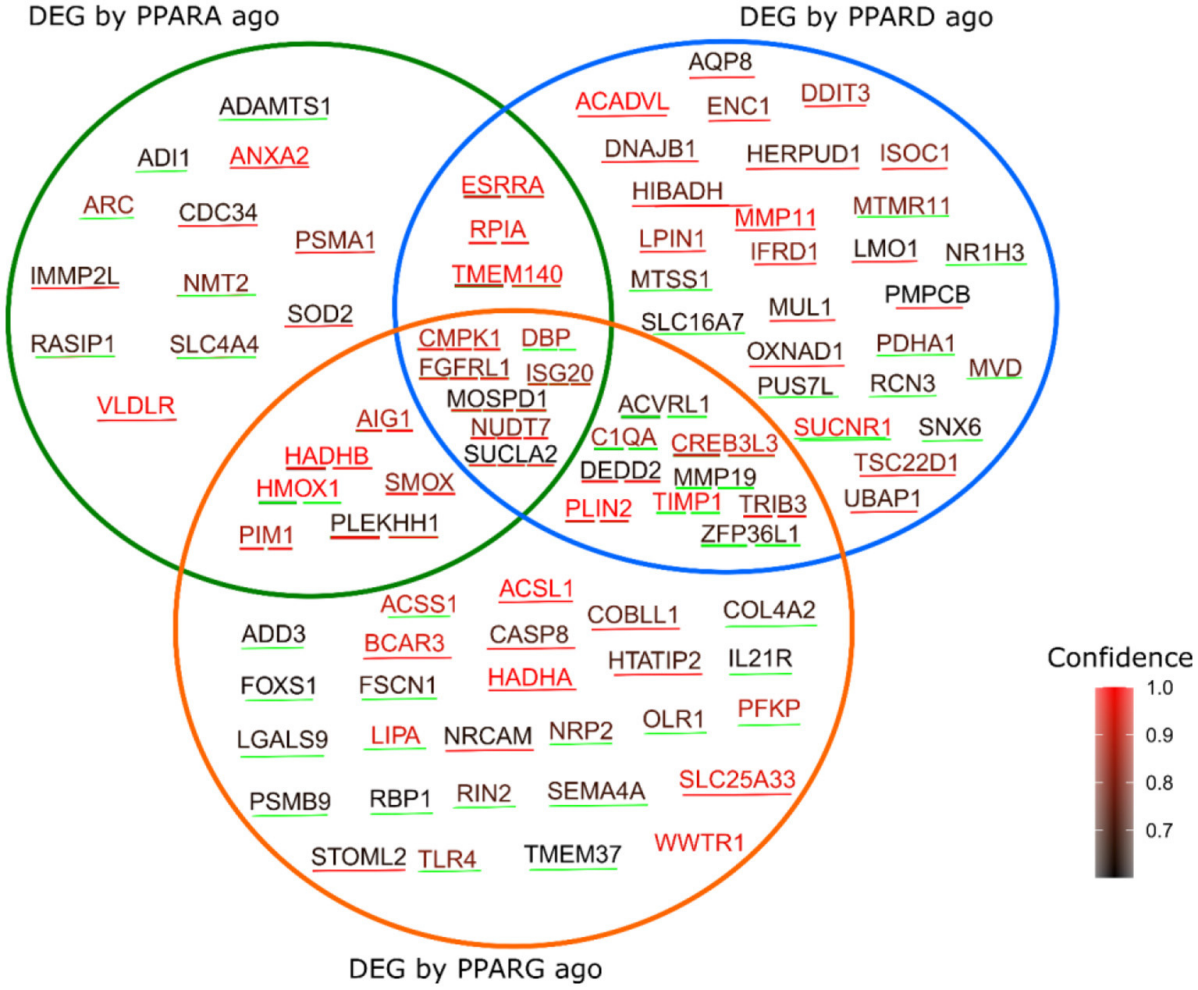
Genes predicted as PPAR target by the PPARgene algorithm, which were also differentially expressed by the three treatments in the current study. The color of the words corresponds to the degree of confidence of the prediction, while the color of the underlining line indicates the direction of the differential expression, with red indicating upregulation in response to the treatment, and green indicating downregulation.

Combining the database in PPARgene with our DEGs, and sub-setting the database to include only values with prediction score > 0.6, reveals a total of 91 predicted targets that were differentially expressed in the three contrasts in our experiment (Figure 7); namely, 12 predicted targets were differentially expressed by the PPARα agonist alone (five were up-regulated), 27 by the PPARδ agonist alone (17 up-regulated), and 27 by the PPARγ agonist alone (eight were up-regulated). Further, three genes were differentially expressed by both the PPARα and PPARδ agonists (all up-regulated), six by both the PPARα and PPARγ agonists (five up-regulated), nine by both the PPARδ and PPARγ agonists (four up-regulated), and seven by all three agonists (six up-regulated).

## Discussion

### Synthetic PPAR Agonists and Antagonists Modulate Very Few Putative PPAR Targets and in the Postpartum Only

A reliable indicator of PPAR activation is the expression of canonical PPAR target genes in response to a putative ligand. Our results indicate a minor modulation of putative PPAR targets in response to PPAR agonists and antagonists in bovine liver PCLS both when cultivated in culture medium or in homologous blood serum. Although we observed minor effects, any effect on the transcription of putative PPAR targets was mostly detectable in post-partum PCLS samples. Prior studies in bovine, which highlight that modulation of PPAR target through supplementation of saturated fatty acids elicits a response primarily in the postpartum, are in line with our results: cows supplemented with saturated fatty acids, proven to be a PPAR ligand in bovine cells (17), displayed an increase in hepatic expression of putative PPAR targets (e.g., *PLIN2, FABP1, ACOX1*) between −14 and +7 days relative to parturition (16). The same design revealed similar outcomes in the adipose tissue, where canonical PPARγ targets were found to be upregulated slightly in the prepartum, but the greatest impact was found in the postpartum in response to either saturated fat or fish oil (rich in unsaturated fatty acids) (41).

Our present data appear to contrast with our prior study, where data indicated a stronger PPAR activation in cultured bovine cells when treated with pre-partum rather than post-partum blood serum (17). Among all genes measured, the *PDK4*, a well-established PPARδ target (42) was consistently affected by the use of synthetic PPARδ agonist or antagonist in the present study confirming results from our prior study using bovine immortalized cells (17, 43). Those data indicated a key role of PPARδ in bovine liver but also confirm that PDK4 is a reliable marker of PPARδ activation in bovine.

The minor effect on the transcription of the RT-qPCR measured putative PPAR target genes in the PCLS treated with the various PPAR agonists may indicate poor response of the liver to PPAR agonist. This is a possibility, considering the poor response observed in prior studies using *in vivo* supplementation of C16:0 on liver transcriptome (16, 44, 45) or the lack of response on P450 enzyme activity to the PPARα agonist Wy-14643 in goats (46). This possibility would be a major obstacle for any nutrigenomic interventions to improve liver performance in dairy cows *via* activation of PPAR, as previously advocated (13).

The genes selected for the RT-qPCR are only putative PPAR targets in bovine, since studies to identify true PPAR isotype specific target genes are still lacking [although the problem has been pointed out for almost a decade (13)]. Thus, it is possible that the relatively minor effect observed through RT-qPCR may be due to an inaccurate choice of target genes on our part. For this reason, we analyzed gene expression at a whole-transcriptome level to assess if there was any response of PCLS to PPAR agonists and, if so, determine PPAR targets in bovine liver.

### Activation of PPAR Does Not Affect TAG in Bovine PCLS

The relatively minor changes in terms of canonical PPAR targets were apparently in line with the minor effect on the amount of TAG in the PCLS. The level of TAG in liver of cows are the combined result of re-esterification of circulating NEFA in TAG that are accumulated as lipid droplets and then released with the VLDL. The synthetic media used in our experiment was supplemented with 10% FBS, containing an overall low amount of free fatty acids (**FA**); thus, the addition of NEFA and C16:0 should have increased accumulation of TAG, if no increase in oxidation was present. On the other hand, the activation of PPARα and PPARδ should have increased oxidation of fatty acids and, perhaps, even increase VLDL secretion (13); thus, we were expecting a decrease in TAG with those treatments.

The role of PPAR in the regulation of hepatic TAG is unclear as prior studies on the topic have revealed. In mice fed a diet deficient in methionine and choline (simulating conditions experienced during non-alcoholic steatohepatitis), administration of the PPARδ agonist GW501516 and the pan-agonist bezafibrate markedly reduced hepatic TAG and lipid droplet size in hepatocytes (47); on the other hand, the same PPARδ agonist failed to reduce liver TAG in mice consuming a “Western type” high-fat diet, though circulating plasma TAG were significantly lower (48). Finally, in mice chronically exposed to carbon tetrachloride (causing fibrosis in the liver), treatment with GW501516 caused a net increase in liver TAG, which was not observed in the PPARδ-knockout population, indicating that activation of PPARδ is required for TAG accumulation under those conditions (49). It is unclear why we observed a tendency for higher TAG accumulation in PCLS treated with PPARδ in our experiment, but we cannot exclude that activation of PPARδ could increase TAG accumulation *in vivo*.

For the PPARα (WY-14643) and PPARγ (rosiglitazone) agonists, the situation is remarkably similar: though most studies report a reduction of plasma TAG (postprandial) when administering PPARα and PPARγ agonist (50–52), conflicting reports exist on the impact on hepatic TAG. In mice fed a high-fat diet, both rosiglitazone and WY-14643, as well as ragaglitazar (dual PPARα/PPARγ agonist) significantly decreased both TAG accumulation and TAG production rates in the liver, with the dual agonist achieving TAG concentrations equal to the control group fed a normal diet (53). In addition, mice exposed to a methionine- and choline-deficient diet (similar to the scenario discussed for the PPARδ agonist above), had a sharp reduction in hepatic TAG after treatment with WY-14643 (54). On the other hand, WY-14643 effected a sharp increase in TAG in primary mouse hepatocytes (55), and induced expression of *HILPDA* in mouse PCLS, the overexpression of which caused a marked decrease in hepatic TAG secretion, and a consequent increase in TAG content within the liver (56). In the present experiment with bovine PCLS, the same transcript was not affected by WY-14643 (Supplementary File 1). Similarly, treatment of genetically diabetic mice with rosiglitazone increased hepatic TAG (57, 58). Other studies have noted that liver-specific expression levels of PPARγ determine the outcome of administering the agonist, as mice with low hepatic *Pparg* expression saw a reduction in TAG accumulation, while mice with high hepatic *Pparg* expression displayed a consistent increase in hepatic TAG (59).

The available evidence seems to suggest that in certain pathophysiological conditions and drastic metabolic alterations, the activation of PPAR (regardless of isotype) can contribute to the clearance of TAG, whereas in metabolically stable animals the outcome is the opposite. It is worth considering that in the studies cited above the agonists were administered for at least 12 days (in the shortest trial) and up to 9 weeks (in the longest); though PCLS are technically viable up to 96 h (28), we noticed significant RNA degradation (RIN < 6) after 24 h of incubation (data not shown). As such, a combination of different metabolic conditions between the animals in our study and those in the cited studies, combined with a markedly shorter incubation time with the agonists, might explain the lack of significant differences in our results.

### Activation of All PPAR Isotypes Induce Pathways Related to Lipid Metabolism

Based on the findings revealed by the DIA analysis, activation of any of the three PPAR isotypes leads to a strong activation of lipid metabolism. The apparent activation of lipid metabolism aligns with the findings of previous investigations. Treatment of liver slices of dairy calves with the PPARα agonist clofibrate led to a significant increase in expression of genes associated with lipid metabolism (60), while an *in vitro* investigation in goat mammary epithelial cells found that activation of PPARδ with the synthetic agonist GW0742 resulted in increased expression of genes associated with fatty acid activation and lipid transport (61). Interestingly, both these studies found significant upregulation of *ACSL1*, the long-chain member of the acyl-coenzyme A synthetase family. Although *PPARG* is typically more abundant in the adipose tissue than in the liver of dairy cattle, we did find a significant upregulation of genes associated with lipid metabolism when the PPARγ agonist rosiglitazone was used as well. Prior work examining PPARγ activity in the liver of ruminants is limited, but activation of PPARγ in the liver of non-ruminants seems to produce a similar upregulation of genes associated with lipid accumulation as is seen in the adipose tissue (62).

### Activation of PPARγ Modulates Pathways Associated With the Immune Response in Liver

Activation of PPARγ resulted in a general inhibition of pathways associated with the immune system. PPARγ has a well-investigated role in modulating the immune response and inflammation (63), with activation of PPARγ limiting the expression of pro-inflammatory cytokines like TNFα (64). Activation of PPARγ in the liver could also impact biliary duct maintenance by regulating intracellular TLR signaling (65).

The inhibition of the immune system revealed by the DIA and DAVID when the liver slices were treated with the PPARγ agonist seems to be driven by several pathways, with the stronger effect observed in the IgA production and antigen processing and presentation. The inhibition of those pathways is mainly driven by the downregulation in transcription coding for major histocompatibility complex-associated proteins involved in the antigen processing and presentation. This effect is likely from the immune cells present in the liver, including Kupfer dendritic cells, Kupffer cells and monocyte-derived myeloid cells that are the most important when considering the antigen processing and presentation in the liver (66). Our tissue culture model likely included all those cells, as indicated by the relatively high RNSA abundance of Kupffer cells markers (Supplementary File 1).

Our results indicate a potential role of activation of PPARγ in reducing inflammation and immune activation in the liver. This can have important practical implications for transition dairy cows, due to the known negative effect of the liver's response to inflammation on performance and liver function in dairy cows (67).

### *In silico* Analysis and Gene Expression Patterns Identifies Novel Putative PPAR Targets in Dairy Cattle

#### Confirmed PPAR Targets

##### Most of Confirmed PPAR Targets Are Involved in Lipid Metabolism

The comparison between PPAR targets predicted by PPARgene and DEG in response to PPAR agonists in this study revealed significant overlap. Some of the identified targets across treatment groups are well-documented PPAR targets: *ACADVL*, involved in the oxidation of very long chain FA, is a known target of PPARδ in monogastrics (68) as well as in cattle (13, 69), and its expression was found to be responsive to NEFA and modulated by metabolic changes in the peripartum (10, 70). In our RNAseq data, *ACADVL* was upregulated in response to the PPARδ agonist. This was not confirmed by the RTqPCR data (see the limitation session). Similarly, perilipin 2 (*PLIN2*), upregulated by both the PPARδ and PPARγ agonists, is a known PPAR target in monogastrics (71), though it is of note that while in monogastrics *PLIN2* is identified as a target of PPARα, studies in goats found its expression to be responsive to rosiglitazone, the same PPARγ agonist used in this study (72). *VLDLR*, the gene encoding the VLDL receptor, was upregulated by the PPARα agonist, and substantial evidence exists indicating it as a target gene of PPARδ (73) and PPARα (74), through which it contributes to lowering serum TAG in mice. Transcription of *LPIN1* was upregulated by the PPARδ agonist in PCLS in our study, the same transcript was previously identified as a PPAR target in bovine cells (13) and several monogastric species (75).

Most of the genes identified as putative targets have a role in the metabolism of lipids and fatty acids, inflammation, the immune response, and the antioxidant system. Of the ones related to lipid and fatty acid metabolism, *ACSS1* and *ACSL1*, encoding acyl-CoA synthetase 2 and acyl-CoA synthetase long-chain, respectively, were differentially expressed in response to the PPARγ agonist rosiglitazone in our study, and are known PPAR targets in monogastrics (76, 77). *ACSL1* in particular was shown to regulate plasma TAG amount through the PPARγ pathway in humans (77); however, these results do not seem to be replicated in prior bovine studies, at least as it pertains to mammary epithelial cells, as the expression of *ACSL1* was not altered by rosiglitazone or several fatty acids (78). Whether modulation of *ACSL1* can be possible and relevant beyond the liver in bovine remains to be determined. Within the same category, *HTATIP2* was upregulated, and *LIPA* and *OLR1* were downregulated in PCLS treated with a PPARγ agonist. *HTATIP2*, which encodes an oxidoreductase, was previously shown to form a complex with acyl-CoA synthase 4 (79), and its overexpression in mouse hepatocytes altered fatty acid metabolism by reducing oxidation rates, and increased esterification as either TAG or cholesteryl esters (80). Additionally, *HTATIP2* expression increased in the liver of ciprofibrate-treated *Cynomolgus* monkeys (81); though ciprofibrate (and fibrates in general) are regarded as PPARα agonists, which would suggest that *HTATIP2* is a PPARα target in monogastric, some evidence exists for the role of ciprofibrate in activating PPARγ at larger doses (82).

*LIPA*, encoding a lysosomal lipase, was previously found to be downregulated in PPARγ-knockout mouse prostate epithelial cells (83), and by overexpression of PPARγΔ5, a recently-discovered isoform of PPARγ, in HEK293 cells (84). Interestingly, mutations in the *LIPA* gene can lead to defects in the storage of cholesteryl esters, which would suggest a plausible link between *LIPA* and *HTATIP2*. Finally, *OLR1*, a receptor for oxidized LDL, was found to be upregulated by rosiglitazone and downregulated in response to the PPARγ antagonist PD068235 in 3T3-L1 mouse adipocytes (85), and its expression was highly correlated with that of PPARγ in porcine adipose tissue (86). An upregulation of the oxidized LDL receptor can lead to increased uptake of oxidized LDL, as well as the accumulation of TAG (86). Downregulation of *OLR1* and *LIPA* suggest that, in the bovine liver, PPARγ agonists may lead to decreased accumulation of TAG; this is further substantiated by the upregulation of *COBLL1* in response to the PPARγ agonist, and of *CREB3L3* by both the PPARγ and PPARδ agonist. In human SGBS preadipocytes, knockout of *COBLL1* increased TAG accumulation by ~30% (87), while *CREB3L3* ablation in mice led to a dramatic increase (~4-fold) in circulating TAG and a consequent increase in hepatic TAG, along with increased ketogenesis (88), though existing evidence in monogastrics highlights a major interaction with PPARα (89), not PPARδ or PPARγ. Our results seem in partial disagreement with these findings, as the amount of TAG in post-partum slices was not significantly different between treatment groups. As mentioned earlier in the discussion, a plausible explanation could be the limited incubation time (~18 h) of the PCLS, suggesting that a longer timeframe may be needed to witness biologically relevant effects.

Among the putative targets, evidence emerges for a role of PPAR agonists in the regulation of FA oxidation through novel mechanisms. *HADHA* and *HADHB*, encoding the subunits of the mitochondrial trifunctional protein MTP, were upregulated by the PPARγ agonist (*HADHA*) and by the PPARα and PPARγ agonist (*HADHB*). MTP is an enzyme associated with the inner mitochondrial membrane, and it catalyzes the final steps of the beta oxidation of long-chain FA in the mitochondria (90). Bezafibrate, a PPARα agonist, upregulated both *HADHA* and *HADHB* in human skin fibroblasts (90), while the PPARα/PPARγ dual agonist LY465608 increased expression of *HADHB* in both rat and dog hepatocytes, simultaneously increasing FA oxidation as measured by acyl-CoA and carnitine palmitoyl transferase I activity (91). Additionally, *NUDT7*, upregulated by all of three PPAR agonists in our study, is known to regulate peroxisomal FA oxidation by modulating coenzyme A degradation (92), and its expression is responsive to PPARα agonist WY-14643 in mouse liver (93). Limited evidence exists for the role of *HADHA/B* and *NUD7* in bovines, most of which is in regards to unrelated production parameters such as meat color (94).

##### Few PPAR Targets Are Coding for Proteins Involved in Antioxidant Response

Two of the putative targets suggest a possible role of PPAR isotypes in regulating the antioxidant response: *SOD2*, encoding mitochondrial manganese superoxide dismutase, is directly regulated by PPARα, and downregulated in PPARα-/– mice (95), and accordingly our results found it upregulated in response to the PPARα agonist. On the other hand, *HMOX1* (hemoxygenase-1) was downregulated by both the PPARα and PPARγ agonists. Studies in rat liver identified a response of *HMOX1* to pioglitazone (PPARγ agonist) (96); this may be a product of indirect regulation, as PPARγ agonist rosiglitazone is known to activate the AMP kinase pathway, of which *HMOX1* is a target (97). To our knowledge, a link between PPAR isotypes and *HMOX1*/*SOD2* has not been elucidated in bovines.

##### PPAR Targets and Insulin Signaling-Related Functions

*DBP*, downregulated by all three PPAR agonists, and *TRIB3*, upregulated by both the PPARδ and PPARγ agonists, are involved in regulating insulin sensitivity. Acetylation of histone 3 lysine 9 at the promoter region of *DBP*, and its consequent upregulation, plays a role in regulation of glucose homeostasis and is related to PPARγ in mice (98) and humans with type 2 diabetes (99), while *TRIB3* affects response to insulin by inhibiting Akt phosphorylation (100), and its activity is directly related to that of PPARγ (101). The differential expression of *TRIB3* observed in response to the PPARδ agonist may be a feature unique to the bovine liver: though a possible link between *TRIB3* expression and PPARα has been established (102), no evidence of an interaction with PPARδ has been elucidated.

##### Confirmed Targets Repressed by PPARγ Are Involved in Inflammation and Immune Regulation

Five genes among the identified putative targets are involved in the regulation of the immune response: *IL21R, LGALS9, SEMA4A, NRP2*, and *TLR4* were all downregulated in the PPARγ agonist group. *IL21R*, encoding an interleukin receptor, is known to play a role in ensuring proper function of T cells in mice (103), and was previously found upregulated by the pan-PPAR agonist elafibranor (104). *LGALS9* (galectin 9) is involved in T cell exhaustion (105), and activates AMPK (106), and was found to be strongly downregulated in response to rosiglitazone (the same PPARγ agonist as in our study) in 3T3-L1 preadipocytes (107). *SEMA4A* encodes semaphorin-4a and its activity is directly dependent on its receptors, neuropilins (of which *NRP2* is one); *SEMA4A* is involved in the proliferation of CD4+ T cells in mice and human (108), and in the function and survival of regulatory T cells in mice (109). Though no direct evidence exists of a link between PPAR and *SEMA4A*, other studies have shown that PPAR agonists can regulate other semaphorins, such as *SEMA6B* [downregulated by all PPAR isotypes in human genomic fragments *in vitro* (110)] and *SEMA3G* [upregulated by PPARγ ligands in human endothelial cells (111)]. Finally, Toll-like receptor 4 (*TLR-4*), also downregulated by the PPARγ in our study, regulates the adaptive immune response by triggering production of pro-inflammatory cytokines. In accordance with our results, rosiglitazone-treated rats displayed lower expression of *TLR4* (112), and human HMEC-1 mammary epithelial cells treated with hypaphorine, which decreased PPARγ expression, had a drastic upregulation of *TLR4* (113).

#### Novel PPAR Targets Are Likely Involved in Regulation of Inflammation

A considerable number of genes related to metalloproteinases (MMP) was observed among the novel putative targets, mostly modulated by the PPARδ and PPARγ agonists. *MMP19* was downregulated in both groups, as well as *TIMP1*, a protein involved in the intracellular regulation of MMP, the abundance of which was markedly lower in rat chondrocytes treated with GW-501516 and rosiglitazone, the same PPARδ and PPARγ agonists used in this study (114). On the other hand, *MMP11* was upregulated in response to the PPARδ agonist in our study. Finally, *ADAMTS1*, a zinc-binding metalloproteinase, was downregulated in response to the PPARα agonist. MMPs have a known role in regulating inflammation, and some evidence of a link with PPAR activity is present in the literature for monogastrics (115, 116). This is certainly the case for *MMP19*, involved in the development of T cells in mice (117); *MMP11*, related to several cytokines in breast cancer cells (118); and *ADAMTS1*, a known regulator of the inflammatory response (119). The concerted modulation of several MMPs in our study suggests an intricated landscape of the inflammatory response as regulated by PPAR.

## Limitations

1) Slice thickness and compound absorption: though the Krumdieck tissue slicer provides relatively consistent precision throughout the slices, prior studies have shown that the thickness of bovine PCLS is remarkably less consistent between slices than that of human, pig, rat and mouse (120). The absorption of small molecules (such as the PPAR agonists used in this manuscript, or C16:0) relies heavily on slice thickness, as the rate of diffusion is limited by the rapid extraction of the compound by the cells in the outer layer (121). A reduction of the slice thickness to 100 μm could ensure that all cells are metabolizing the compound (121); however, the likelihood of obtaining brittle tissue (especially in the inconsistency-prone bovine PCLS) increases greatly, rendering the endeavor practically impossible. Future studies may benefit from the use of more accurate machines in the preparation of PCLS, such as the Leica VT1200 S (120).
2) Cellular composition of PCLS: utilizing liver slices ensures that the histology and morphology of the tissue is maintained; however, obtaining liver samples through liver biopsy does not guarantee homogenous composition of each sample, as the operator cannot visually identify the lobes of the liver prior to puncture. Though these differences are bound to be minor, two slices proceeding from different areas of the liver are likely to display different patterns of gene expression. In future studies, the use of ultrasonographic examination to determine liver morphology prior to sampling may allow to obtain more consistent results.
3) Statistical power: the study had a relatively low sample size (originally four animals but in the final analysis only three animals, especially for the RNAseq dataset). This led to large variability between biological replicates, especially in the prepartum, where the range of DIM of the four samples was rather large (prepartum samples were collected based on expected calving dates, which can be an imprecise estimate for some animals). The reason for such limitation is 2-fold: one was financial, the utilization of different treatments resulted in a relatively large number of samples to be sequenced reaching the maximum financial allowance for such analysis; the other reason was the attempt to minimize the use of animals. To partially addressed those issues and minimize technical variation, samples from each animal were run in duplicate *in vitro* and post-treatment duplicates were pooled, as indicated above. Future studies will benefit from a more limited range of treatments and larger replication for each group, which will undoubtedly be more feasible considering the rapidly declining costs of NGS technologies.
4) Breed and parity: our study included only primiparous Jersey cows. Other breeds and multiparous cows may have a different response to our experimental setup. For example: Holstein cows have a greater loss of body condition in the peripartum, as well as higher NEFA and BHBA postpartum, and overall higher negative energy balance (122–124). All of these would like impact the response of PCLS to PPAR activation. The present study is meant to be a starting point for further exploration of PPAR activation in bovines using PCLS; future studies will undoubtedly benefit from including other breeds and test multiparous cows.
5) To optimize the use of the limited funding available to achieve the stated objective, many samples (60) were sequenced in each lane. Though the library prep kit used is apt at maximizing sequencing depth when compared with other commercial solutions, we noticed that many transcripts with (typically) a relatively low expression were either not detected or detected at very low level, with zero-counts in most samples. Despite being able to detect >14,000 transcripts with a count>4 in at the least one sample, which is somewhat similar to what previously reported in RNAseq analysis of bovine liver (125–127), many transcripts with a relatively medium-low expression were either not detected or detected at very low level, with lack of detection in most samples. Most of the genes used for the RT-qPCR were in this category, such as all PPAR transcripts and *LIPC*. Other were undetectable in RNAseq, such as *PPARGC1A* and *HES6*. Among the transcripts detected in all the samples (i.e., *PDK4, ACADVL*, and *FABP1*), a positive correlation was observed between results of RTqPCR and RNAseq when both were normalized using reference genes (Supplementary File 2). The low depth likely limited the discovery of other PPAR target genes.

## Conclusions

Bovine PCLS are responsive to treatment with PPAR agonists and reveal a complex and heterogeneous transcriptomic response. Though minimal changes in TAG accumulation were detected, suggesting no effective changes in oxidation and esterification rates, several genes involved in lipid metabolism were altered in response to the PPAR agonists in the postpartum, suggesting that the lack of enzymatic effect may be due to the relatively short incubation time. An important suggestion from our study is a relationship between PPARγ activation and potential decreased inflammation in the liver, that can tremendously benefit post-partum cows. Of the differentially expressed genes identified in the study, a considerable number of them corresponds to putative PPAR target genes, predicted *in silico*; further, many of these find support in the literature, as prior studies highlight their link with PPAR activation. In summary, PCLS represent a valuable model of the bovine liver of periparturient dairy cows

## Data Availability Statement

Raw sequencing reads have been deposited to NCBI Gene Expression Omnibus (GEO accession number GSE183063). All relevant processed data is provided in the Supplementary Materials. Any additional documents will be provided by the authors upon request.

## Ethics Statement

The animal study was reviewed and approved by Institutional Animal Care and Use Committee (IACUC) of Oregon State University (protocol# 4894).

## Author Contributions

SB helped performing liver biopsy, collected blood, optimized the PCLS, performed all the experiments, analyzed the data, interpreted the data, and wrote the manuscript. HF helped interpreting the data and revised the manuscript. AA performed RT-qPCR analysis and revised the manuscript. CE performed liver biopsies and revised the manuscript. MB received the funding, helped in interpreting the data, helped writing the manuscript, and revised the final manuscript. All authors contributed to the article and approved the submitted version.

## Funding

This project was funded by the Oregon Beef Council.

## Conflict of Interest

The authors declare that the research was conducted in the absence of any commercial or financial relationships that could be construed as a potential conflict of interest.

## Publisher's Note

All claims expressed in this article are solely those of the authors and do not necessarily represent those of their affiliated organizations, or those of the publisher, the editors and the reviewers. Any product that may be evaluated in this article, or claim that may be made by its manufacturer, is not guaranteed or endorsed by the publisher.

## Supplementary Material

Supplementary File 1 Complete dataset with annotation and statistical results.

Supplementary File 2 Comparison between RTqPCR and RNAseq for selected transcripts.

Supplementary Figure S1 Principal component analysis of the RNAseq.

Supplementary Figure S2 Enriched GO terms within the cohort of genes significantly upregulated (FDR-adjusted *p*-value <0.2) by treatment with the PPARα agonist.

Supplementary Figure S3 Enriched GO terms within the cohort of genes significantly downregulated (FDR-adjusted *p*-value <0.2) by treatment with the PPARα agonist.

Supplementary Figure S4 Enriched GO terms within the cohort of genes significantly upregulated (FDR-adjusted *p*-value <0.2) by treatment with the PPARδ agonist.

Supplementary Figure S5 Enriched GO terms within the cohort of genes significantly downregulated (FDR-adjusted *p*-value <0.2) by treatment with the PPARδ agonist.

Supplementary Figure S6 Enriched GO terms within the cohort of genes significantly upregulated (FDR-adjusted *p*-value <0.2) by treatment with the PPARγ agonist.

Supplementary Figure S7 Enriched GO terms within the cohort of genes significantly downregulated (FDR-adjusted *p*-value <0.2) by treatment with the PPARγ agonist.

Supplementary Table S1 Accession number, gene symbol, sequence, and amplicon size of quantified genes, and internal control genes. If reference is lacking, primers were designed for this manuscript.
