# Supplementary figures and images for: Peroxisome Proliferator-Activated Receptor Activation in Precision-Cut Bovine Liver Slices Reveals Novel Putative PPAR Targets in Periparturient Dairy Cows
Source: Front Vet Sci. 2022 Jul 12;9:931264. doi: 10.3389/fvets.2022.931264 (PMC9315222; doi:10.3389/fvets.2022.931264)

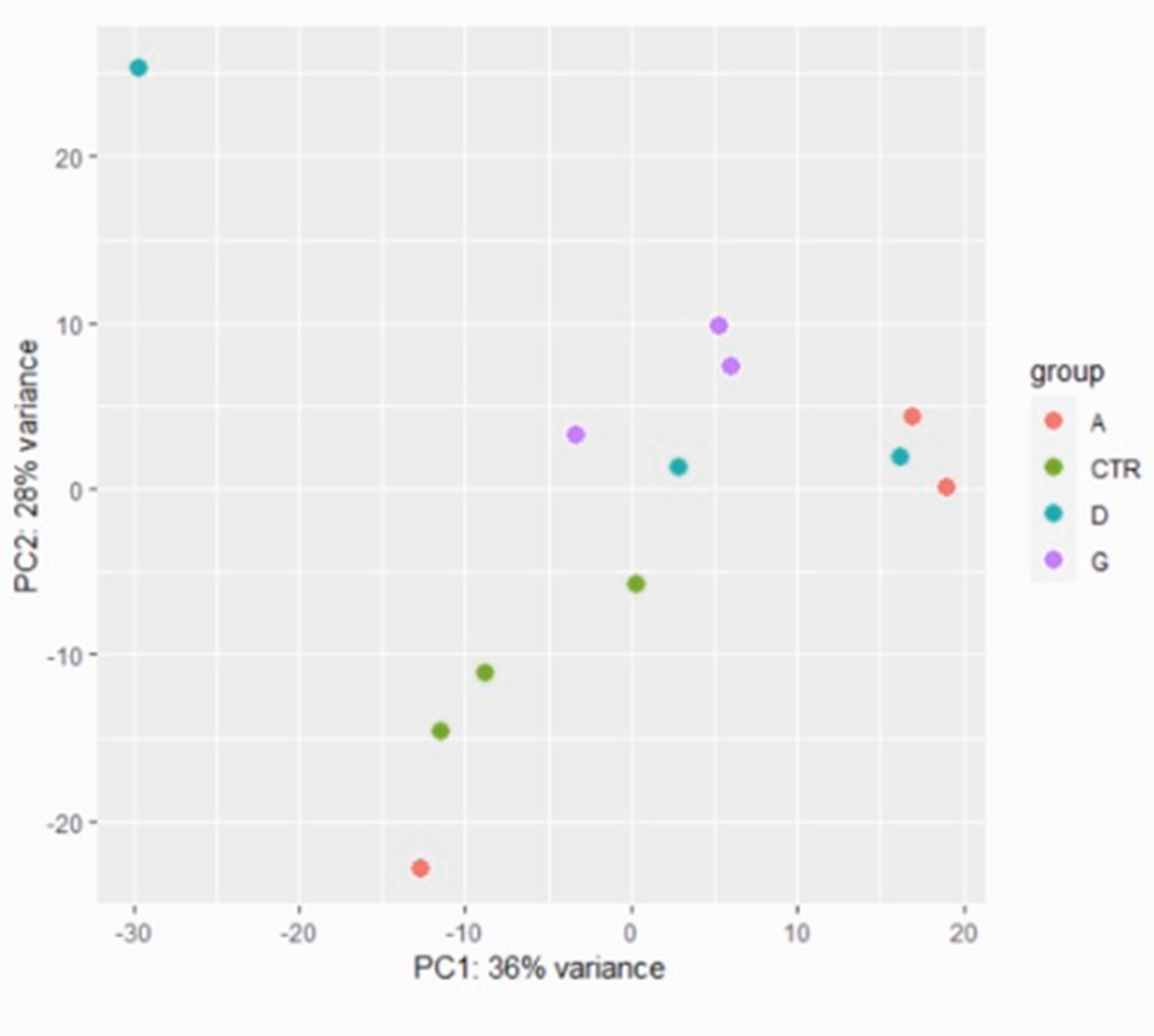

Supplement: Supplementary Figure S1 — Principal component analysis of the RNAseq. [file Image_1.TIFF]

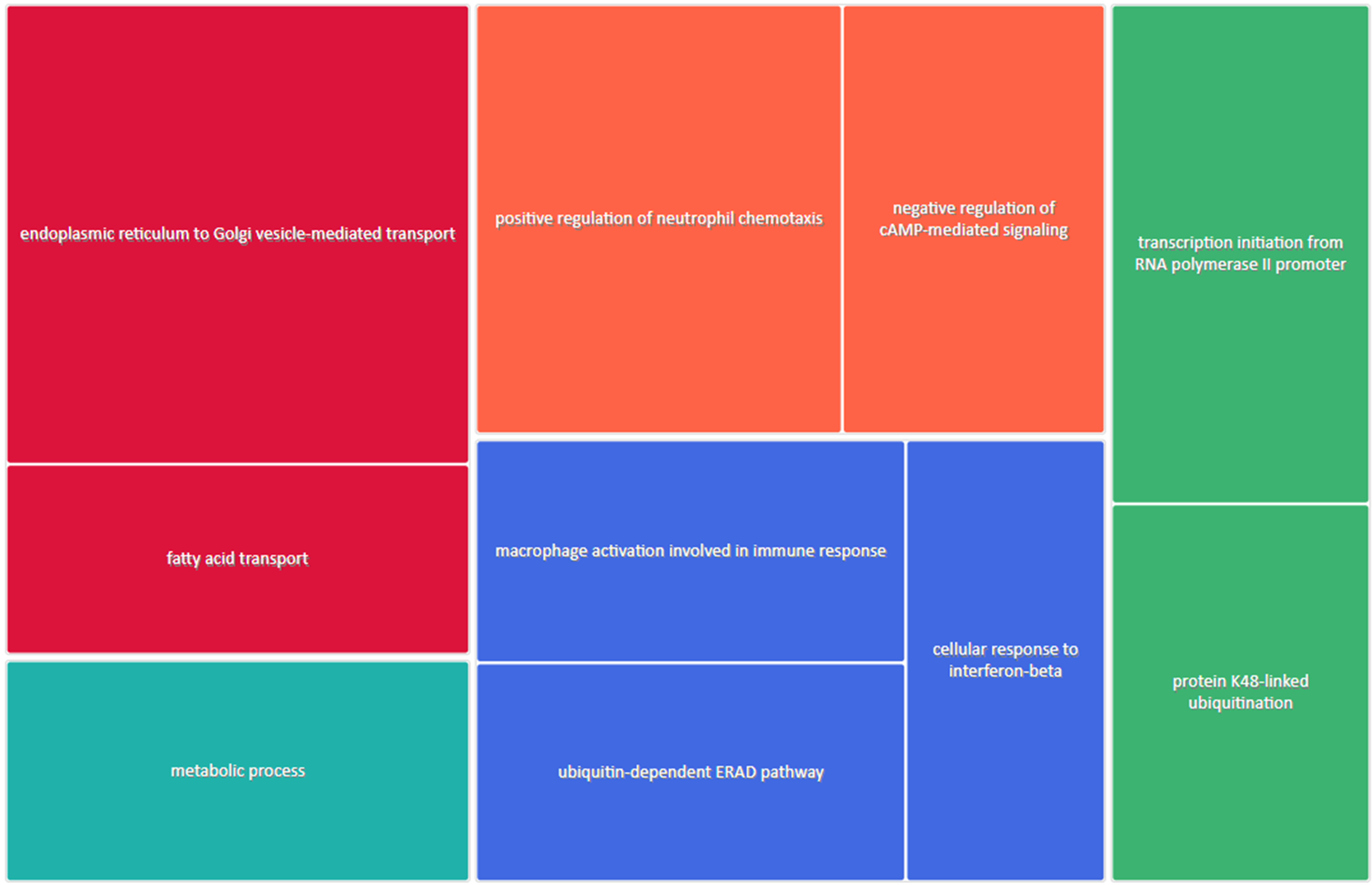

Supplement: Supplementary Figure S2 — Enriched GO terms within the cohort of genes significantly upregulated (FDR-adjusted p-value <0.2) by treatment with the PPARα agonist. [file Image_2.TIF]

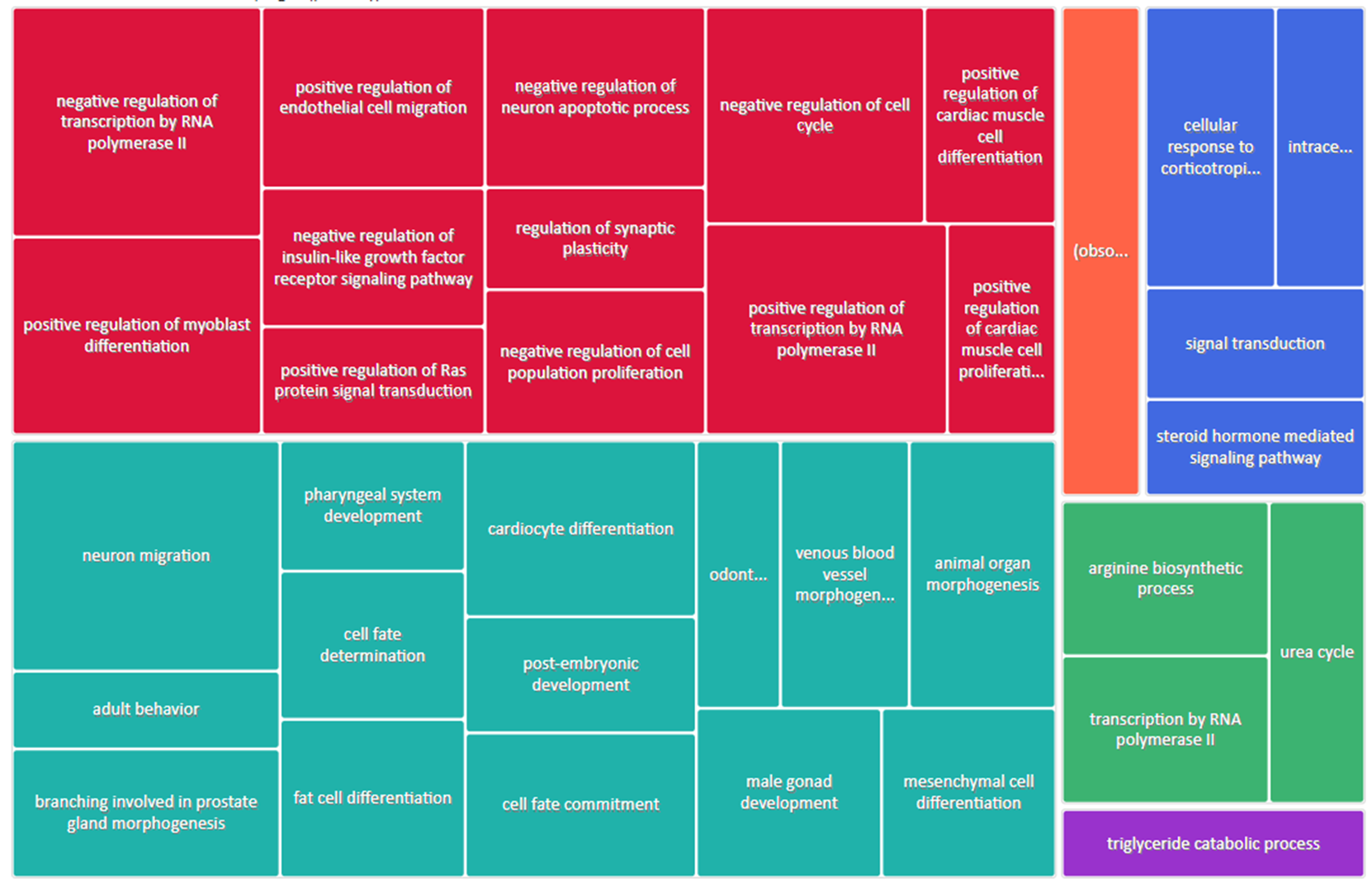

Supplement: Supplementary Figure S3 — Enriched GO terms within the cohort of genes significantly downregulated (FDR-adjusted p-value <0.2) by treatment with the PPARα agonist. [file Image_3.TIF]

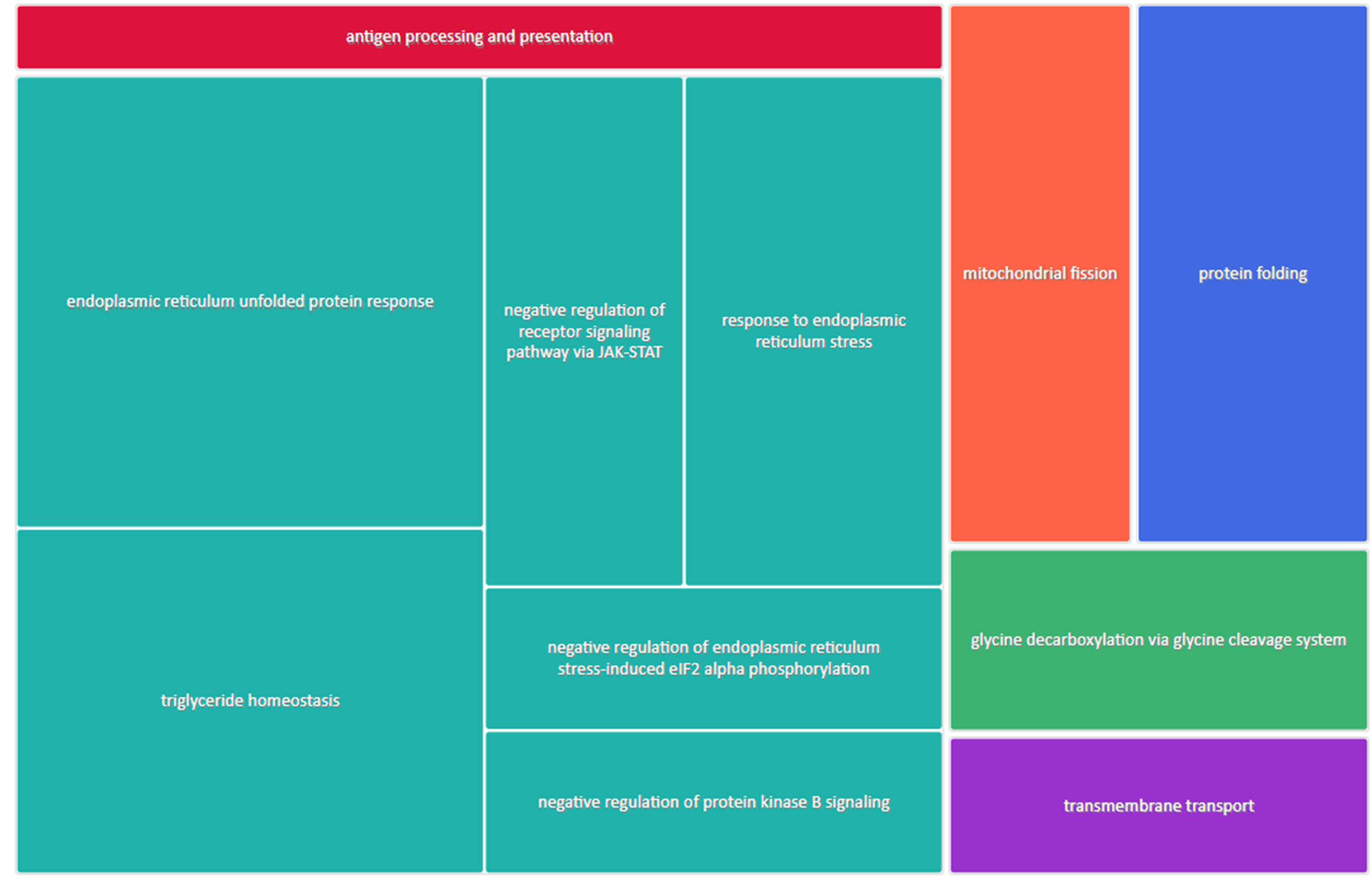

Supplement: Supplementary Figure S4 — Enriched GO terms within the cohort of genes significantly upregulated (FDR-adjusted p-value <0.2) by treatment with the PPARδ agonist. [file Image_4.TIF]

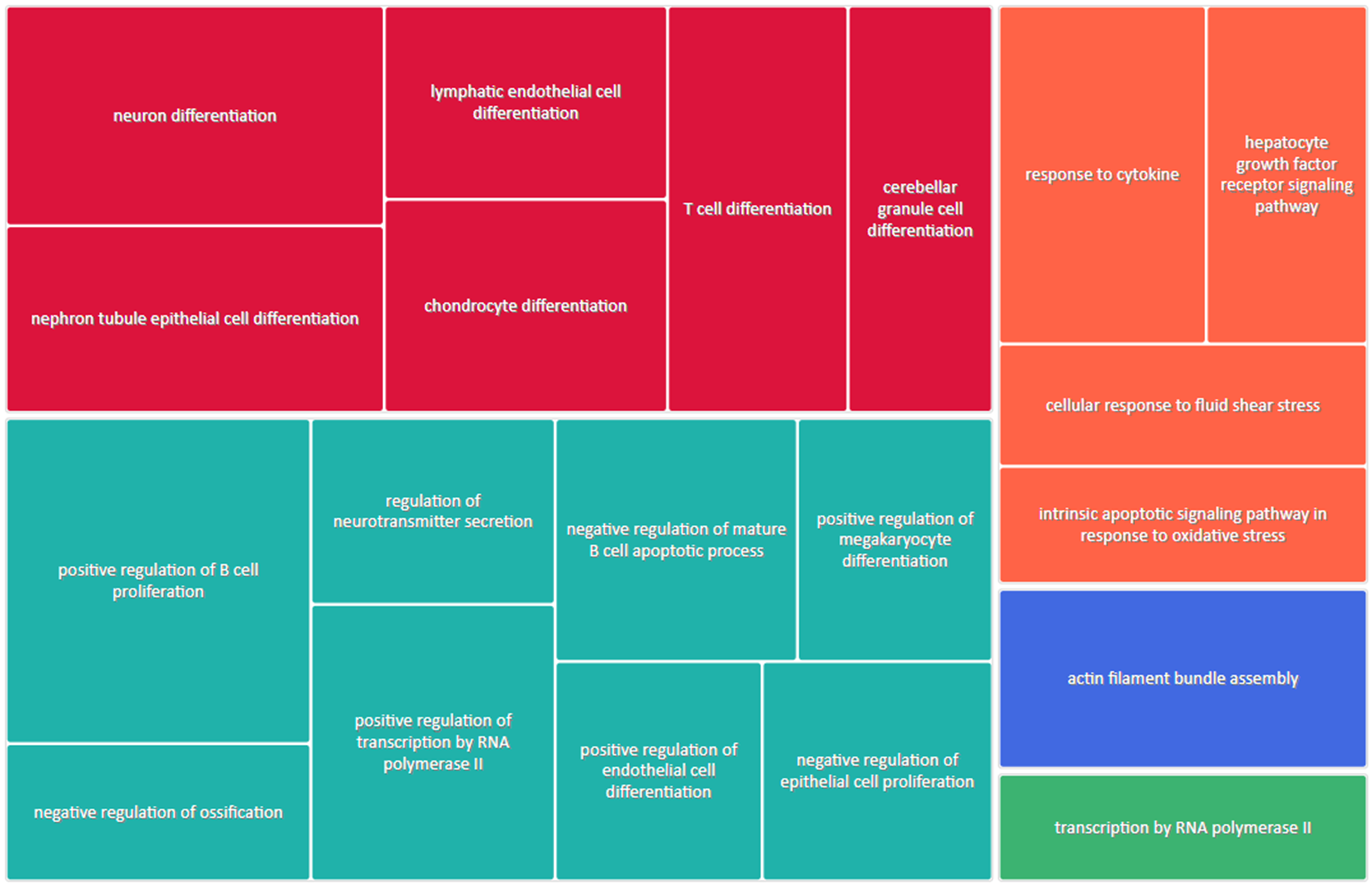

Supplement: Supplementary Figure S5 — Enriched GO terms within the cohort of genes significantly downregulated (FDR-adjusted p-value <0.2) by treatment with the PPARδ agonist. [file Image_5.TIF]

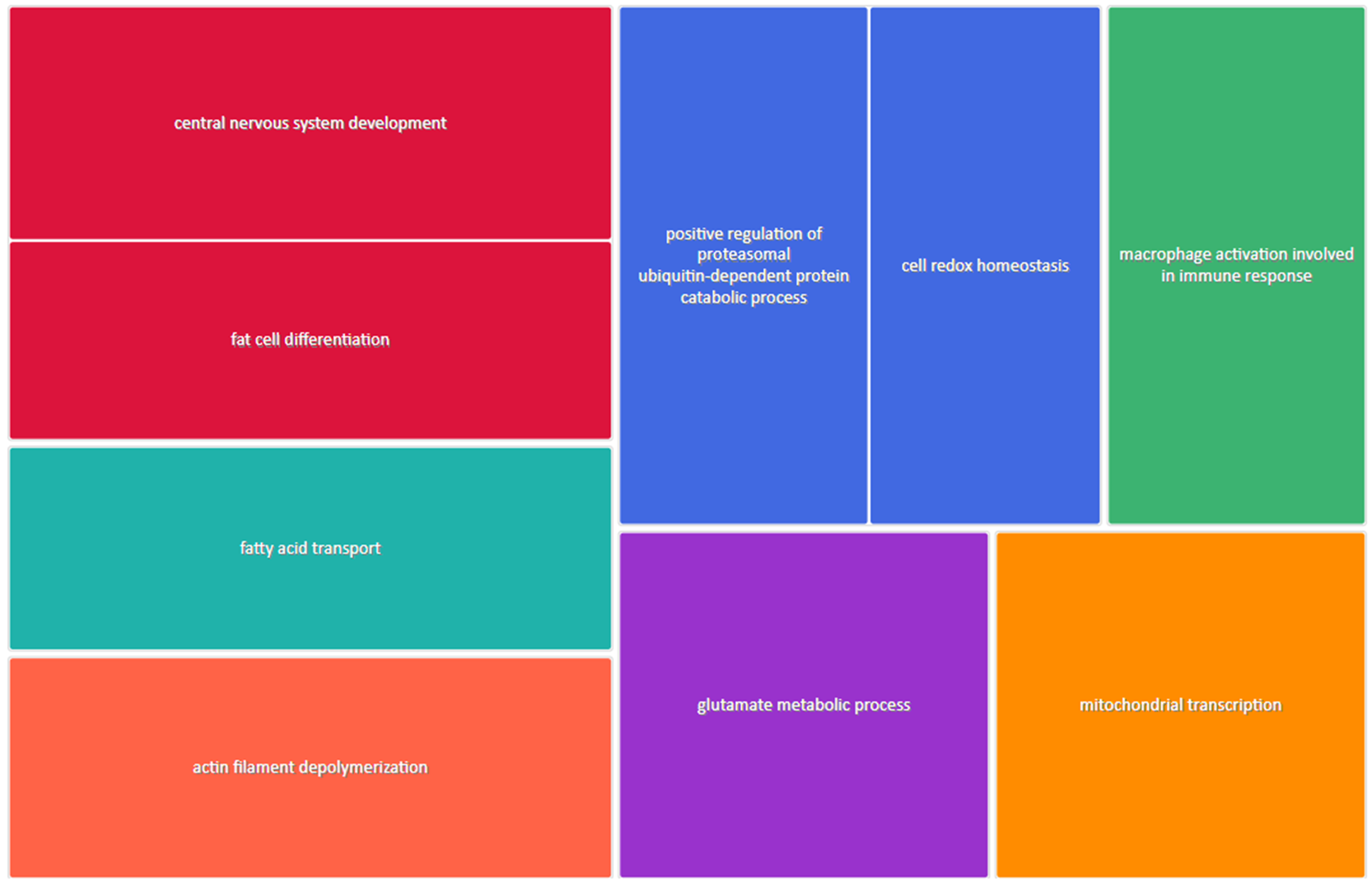

Supplement: Supplementary Figure S6 — Enriched GO terms within the cohort of genes significantly upregulated (FDR-adjusted p-value <0.2) by treatment with the PPARγ agonist. [file Image_6.TIF]

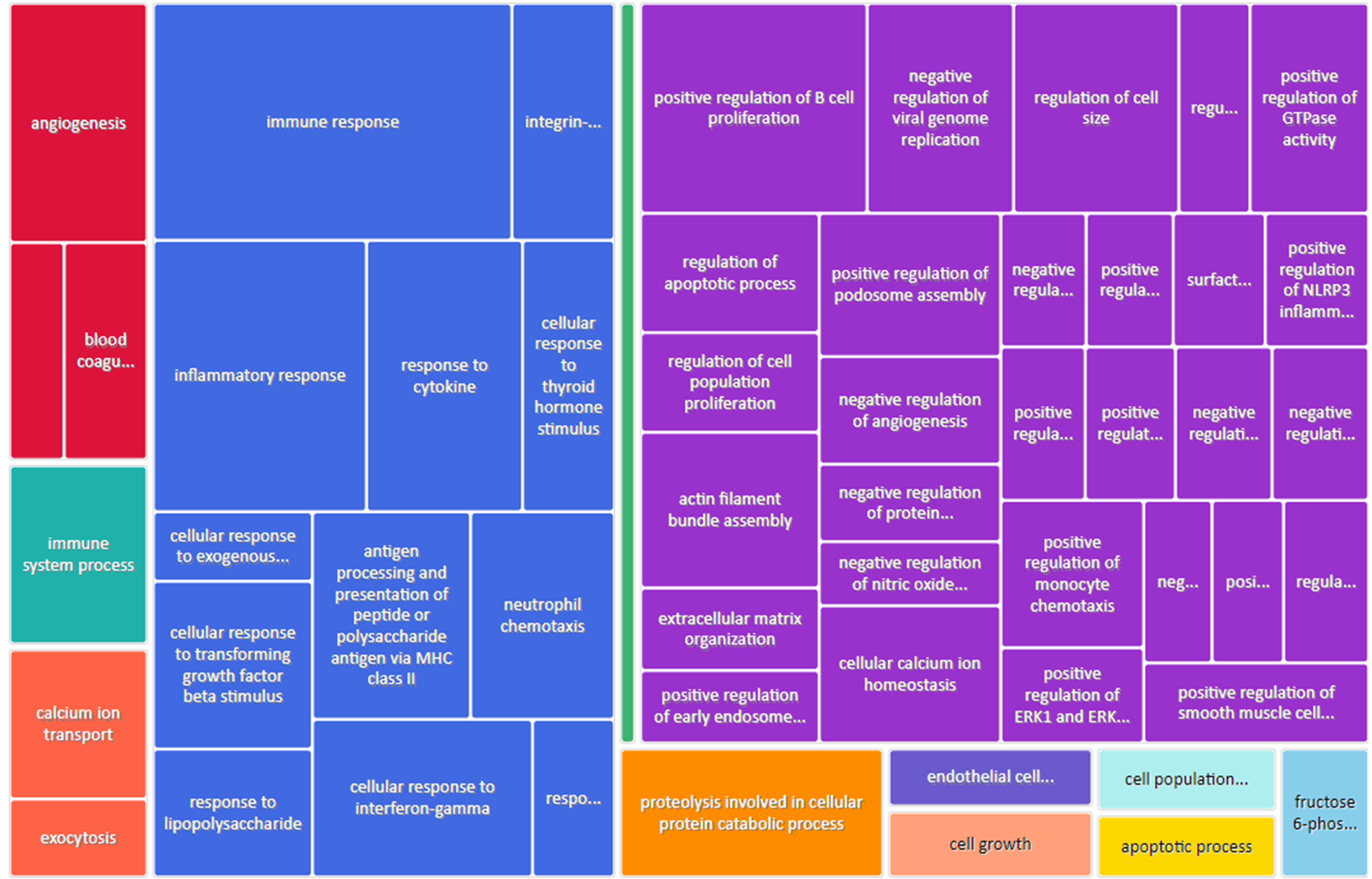

Supplement: Supplementary Figure S7 — Enriched GO terms within the cohort of genes significantly downregulated (FDR-adjusted p-value <0.2) by treatment with the PPARγ agonist. [file Image_7.TIF]
