# Supplementary material for: Peroxisome Proliferator-Activated Receptor Activation in Precision-Cut Bovine Liver Slices Reveals Novel Putative PPAR Targets in Periparturient Dairy Cows
Source: Front Vet Sci. 2022 Jul 12;9:931264. doi: 10.3389/fvets.2022.931264 (PMC9315222; doi:10.3389/fvets.2022.931264)
Supplement: Supplementary Table S1 — Accession number, gene symbol, sequence, and amplicon size of quantified genes, and internal control genes. If reference is lacking, primers were designed for this manuscript. [file Table_1.DOCX]

**Table S 1.** Accession number, gene symbol, sequence, and amplicon size of quantified genes, and internal control genes. If reference is lacking, primers were designed for this manuscript.

| Gene Symbol | Accession | Hybridization | Sequence (5'->3') | Amplicon Size | Reference |
| --- | --- | --- | --- | --- | --- |
| *ACADVL* | NM_174494.2 | F.707 | CCAGCCCCTGTGGAAAATACTA | 62 |  |
|  |  | R.768 | GCCCCCGTTACTGATCCAA |  |  |
| *FABP1* | NM_175817.3 | F.182 | GTTCATCATCACCGCTGGCT | 101 |  |
|  |  | R.282 | CCACTGCCTTGATCTTCTCCC |  |  |
| *HES6* | NM_001075949.1 | F.811 | GAACCACCTGCTCGAGTCC | 119 |  |
|  |  | R.929 | CACCTCCTACGAGCCAGTTG |  |  |
| *LIPC* | NM_001035410.2 | F.1405 | GGGAGAAACACAGCAAAGAATGAC | 100 |  |
|  |  | R.1504 | GTTTGCTTCACATCTCACAAAGGT |  |  |
| *PDK4* | NM_001101883.1 | F.942 | ATGTTCCATCTCACCTTCACCAT | 65 | [169] |
|  |  | R.1006 | AACTGTGGCCCTCATTGCAT |  |  |
| *PGC1A* | NM_177945.3 | F.496 | GCAGAAGAGCCGTCTCTACTTAAGA | 101 |  |
|  |  | R.596 | TTTGCATGGTTCTGGGTACTGA |  |  |
| *PPARA* | NM_001034036.1 | F.729 | CATAACGCGATTCGTTTTGGA | 102 |  |
|  |  | R.830 | CGCGGTTTCGGAATCTTCT |  |  |
| *PPARD* | NM_001083636.1 | F.460 | TGTGGCAGCCTCAATATGGA | 100 | [169] |
|  |  | R.559 | GACGGAAGAAGCCCTTGCA |  |  |
| *PPARG* | NM_181024.2 | F.135 | CCAAATATCGGTGGGAGTCG | 101 |  |
|  |  | R.253 | ACAGCGAAGGGCTCACTCTC |  |  |
| *GAPDH* | NM_001034034 | F.908 | TTGTCTCCTGCGACTTCAACA | 103 | [246] |
|  |  | R.1010 | TCGTACCAGGAAATGAGCTTGAC |  |  |
| *MRPL39* | NM017446 | F.493 | AGGTTCTCTTTTGTTGGCATCC | 101 | [246] |
|  |  | R.593 | TTGGTCAGAGCCCCAGAAGT |  |  |
| *UXT* | NM_001037471 | F.134 | CAGCTGGCCAAATACCTTCAA | 125 | [246] |
|  |  | R.288 | GTGTCTGGGACCACTGTGTCAA |  |  |
